# Health-related quality of life of informal carers in ALS: a systematic review of person reported outcome measures

**DOI:** 10.1007/s11136-025-04012-y

**Published:** 2025-06-25

**Authors:** Rosie Bamber, Theocharis Stavroulakis, Christopher McDermott, Jill Carlton

**Affiliations:** 1 Division of Neuroscience, School of Medicine and Population Health, Sheffield Institute for Translational Neuroscience (SITraN), University of Sheffield, 385A Glossop Road, S10 2HQ Sheffield, U.K.; 2 Sheffield Centre for Health and Related Research (SCHARR), School of Medicine and Population Health, University of Sheffield, Regent Court, 30 Regent Street, S1 4DA Sheffield, U.K.

**Keywords:** Amyotrophic lateral sclerosis, Motor neuron disease, COSMIN, Caring, Carers, Quality of life, Health-related quality of life, Content Validity, Systematic review, Person reported outcome measure

## Abstract

**Purpose:**

Amyotrophic Lateral Sclerosis (ALS) is a fatal neurodegenerative condition with swift progression. The devastating impact of ALS affects the health-related quality of life (HRQoL) of informal carers. Various person reported outcome measures (PROMs) have been used to assess HRQoL in informal carers in ALS, yet their validity remains unclear. This review aimed to identify and evaluate the content validity of HRQoL PROMs for informal carers in ALS.

**Methods:**

This review was conducted according to best practice COnsensus-based Standards for the selection of health Measurement INstruments (COSMIN) methodology. Two literature searches were conducted in November 2023 and April 2024 across MEDLINE, PsycINFO, Embase, CINAHL, the Cochrane Database of Systematic Reviews, CENTRAL and Google Scholar, to identify HRQoL PROMs used with informal carers in ALS, PROM development articles, and psychometric literature. Evidence synthesis followed COSMIN guidance.

**Results:**

12,276 articles were screened, and 109 PROMs were identified, with 43 undergoing full COSMIN assessment. Content validity ratings were ‘*Inconsistent*’ or ‘*Insufficient*’ for all PROMs. All PROMs, except the CarerQoL, were rated ‘*Insufficient*’ for comprehensiveness. Only 18.6% of PROMs included informal carers in development. Quality of evidence supporting content validity ratings was ‘*Very Low*’ for 93% of PROMs.

**Conclusion:**

HRQoL PROMs used with informal carers in ALS lack evidence to support their content validity, restricting their utility for this purpose. Existing literature on the impact of caring in ALS on informal carers’ HRQoL should be interpreted cautiously. Further research is required to establish the content validity of HRQoL PROMs used for this cohort.

**Supplementary Information:**

The online version contains supplementary material available at 10.1007/s11136-025-04012-y.

## Abstract (plain language summary)

Amyotrophic Lateral Sclerosis (ALS) is an incurable condition that worsens quickly and leads to death. It has a huge impact on the quality of life of those who provide care to someone with ALS. Various questionnaires have been used to measure quality of life in informal carers in ALS, but it is unclear whether these questionnaires truly reflect carers’ experiences. This review examines whether these questionnaires are suitable for measuring informal carers’ quality of life in ALS. Our findings show that there is insufficient evidence for the use of existing questionnaires with ALS carers, which restricts their usefulness in clinical and research contexts. As a result, current research that reports experiences of ALS carers using existing questionnaires is limited and should be interpreted with caution.

**Supplementary Information:**

The online version contains supplementary material available at 10.1007/s11136-025-04012-y.

## Introduction

Amyotrophic lateral sclerosis (ALS) is a heterogenous adult-onset neurodegenerative condition characterised by loss of motor neurons in the motor cortex, brainstem and spinal cord, leading to progressive muscle weakness and wasting [1]. In the absence of curative treatment, symptomatic care is offered to prolong life and optimise health-related quality of life (HRQoL) [2]. Respiratory failure is typically the cause of death in ALS, with median survival from onset ranging from 20 to 48 months [3]. ALS has a pooled worldwide incidence of 1.75-3 per 100,000 persons per year, however, significant geographical variation exists [4]. Non-motor features of ALS are now increasingly recognised, with ALS understood as a multi-system disease spectrum from pure motor ALS (50%) to ALS with frontotemporal dementia (FTD) (15%) [5].

Complex and rapidly progressive motor and non-motor symptomatology in ALS place demands on informal carers, who provide unpaid care support [6]. Internationally, informal care in ALS is commonly provided at home by a spouse or close family member [7] who frequently have no prior caregiving experience [8]. As an incurable and often rapidly disabling condition, ALS is recognised to impact the HRQoL of both those living with the condition and their informal carers. Carers may experience significant psychological distress [6, 9]physical burden [10]social isolation [11]and financial hardship [12].

The concept of HRQoL is subjective, lacking a universally accepted definition. For the purposes of the current research, HRQoL is defined as the effect of health state on psychological, physical and social domains of function [13]. The subjectivity inherent to the concept of HRQoL poses ongoing challenges in its measurement, which can occur through qualitative [14] or quantitative [15] methods. Various Person Reported Outcome Measures (PROMs) have been employed to quantitatively assess informal carers’ HRQoL in ALS. Some PROMs are generic and are designed for use across different conditions (e.g. EQ-5D-5L [16]), whilst others are disease- (e.g. ALS Functional Rating Scale Revised [17]) or population-specific (e.g. CarerQoL [18]). Some PROMs selectively measure one HRQoL domain, such as the ALSFRS-R [17] that explores physical functioning; whilst others encompass all HRQoL domains (e.g. Short Form-36 [19]).

Studies have used PROMs to explore informal carer HRQoL in ALS [6, 20]however, they lack an evaluation of the content validity of these PROMs for this cohort. With numerous PROMs available, it is challenging to determine their suitability for assessing carer HRQoL in ALS without this evidence. To date, no reviews have specifically investigated the content validity of HRQoL PROMs for informal carers in ALS. Historically, outcome measurement research in ALS has focused on the experiences and needs of people living with the disease, with comparatively less attention given to their informal caregivers. Choice of PROM(s) should be based on robust evidence of psychometric properties for a specific target population and context (i.e. informal carers in ALS).

Content validity can be described as the extent to which the content of a PROM adequately reflects the construct of interest [21] and is considered to be the most important psychometric property by the COnsensus-based Standards for the selection of health Measurement INstruments (COSMIN) [22]. It can be further conceptualised by considering three key features: (1) relevance, (2) comprehensiveness and (3) comprehensibility (i.e. understanding). Relevance considers whether PROM items (questions), response options and recall period are relevant for the construct (HRQoL), target population (informal carers of people living with ALS) and context; comprehensiveness considers whether a PROM comprehensively encompasses all key aspects of the construct; and comprehensibility considers whether the content of the PROM is understood by the target population [22].

COSMIN methods are recognised as international best practice, to provide a systematic way to evaluate the quality of evidence for the content validity of PROMs to improve their selection in research and clinical contexts [23]. COSMIN methodology is increasingly used to evaluate the validity of PROMs and has been used to evaluate PROMs used in neurological conditions [24, 25]. The aim of the current review is therefore to identify and evaluate the content validity of HRQoL PROMs for informal carers of people living with ALS. The following objectives support achievement of this aim: (1) to identify which HRQoL PROMs have been used with informal carers of people with ALS; (2) to establish the strength and quality of evidence for the content validity of the PROMs identified for assessing HRQoL in informal carers of people with ALS.

## Methods

The review was conducted according to COSMIN guidelines [22, 23] and was reported according to the guideline for systematic reviews of outcome measurement instruments (PRISMA-COSMIN for OMIs) [26]. This review follows a protocol registered in the International Prospective Register of Systematic Reviews (Registration Number: CRD42023484037 [27]).

### Search strategy

An information specialist was consulted in developing a comprehensive search strategy across seven databases (Medical Literature Analysis and Retrieval System Online [MEDLINE], PsycINFO, Excerpta Medica Database [Embase], Cumulative Index to Nursing and Allied Health Literature [CINAHL], the Cochrane Database of Systematic Reviews, the Cochrane Central Register of Controlled Trials [CENTRAL] and Google Scholar), utilising specific database combinations for systematic reviews [28]. Syntax was tailored per database and no restrictions were applied to publication date. This review involved two searches. Search 1, conducted on November 24, 2023, identified PROMs used to measure HRQoL (or an aspect of) in informal carers in ALS using related search terms. Search 2, conducted on April 4, 2024, sourced PROM development articles and literature on the measurement properties of the PROMs identified from Search 1, using related search terms and identified PROMs. Search terms in the current review included: (1) ALS (and derivatives); (2) a comprehensive list of informal carer terms; (3) a comprehensive search filter to identify questionnaires developed by the PROM Group at the University of Oxford [29]; (4) names of PROMs identified in Search 1; (5) a search filter developed by the COSMIN group for identifying articles reporting measurement properties of PROMs [30]. Additionally, as recommended by COSMIN methodology and consistent with other COSMIN reviews [31, 32]supplementary searches were conducted by screening the first 100 Google Scholar results for the names and acronyms of these PROMs. Finally, manual searches were conducted for PROM development articles. All searches were conducted by primary researcher (RB). Supplementary Material 1 details the review search strategy.

### Article screening

Article screening was conducted independently by two researchers (RB and JC) following predefined eligibility criteria (Table 1) using a hierarchical screening tool [33]. Search results were imported into EndNote 21 (Clarivate Analytics) to support a systematic, reproducible deduplication strategy [34]. Following deduplication, search results were transformed into Microsoft Excel (Microsoft Office, V.16.16.27) for title and abstract screening prior to full-text review. For title and abstract screening, one researcher (RB) reviewed all eligible articles, whilst a second researcher (JC) reviewed a random sample of 20% of titles and abstracts. Where disagreement occurred, this was resolved through retaining an article for full-text screening. For full text screening, two researchers (RB and JC) independently reviewed 100% of articles. Any discrepancy was resolved through discussion and reasons for exclusion were documented.

**Table 1:** Article inclusion and exclusion criteria. ALS = Amyotrophic lateral sclerosis, HRQoL = Health-Related quality of life, prom = person reported outcome measure. *Pertains to inclusion criteria applied in search 2 only

Inclusion	Exclusion
• **Subjects**: Adult informal carers (≥ 18) of individuals with ALS. No restrictions to race, ethnicity, geography, or socioeconomic status. • **Intervention/ Exposure**: Assessment via a multi-item, freely available, self-report PROM measuring HRQoL or a domain of HRQoL. • **Outcome**: HRQoL measurement. • **Articles**: Primary research, published as a full-text original article in English, that uses a freely available, multi-item self-report HRQoL PROM with adult informal carers of people with ALS. *Reports data on the content validity of the HRQoL PROM/s identified and used for review of informal carers of people living with ALS. *Qualitative or quantitative development articles of HRQoL PROMs identified from Search 1.	• Articles without available full text (e.g., published abstracts). • Articles including informal carers of mixed syndromic groups, unless the carer population include more than 75% of informal carers of people with ALS, or separate data is available for informal carers of people with ALS.

### PROM screening

**Fig. 1:**
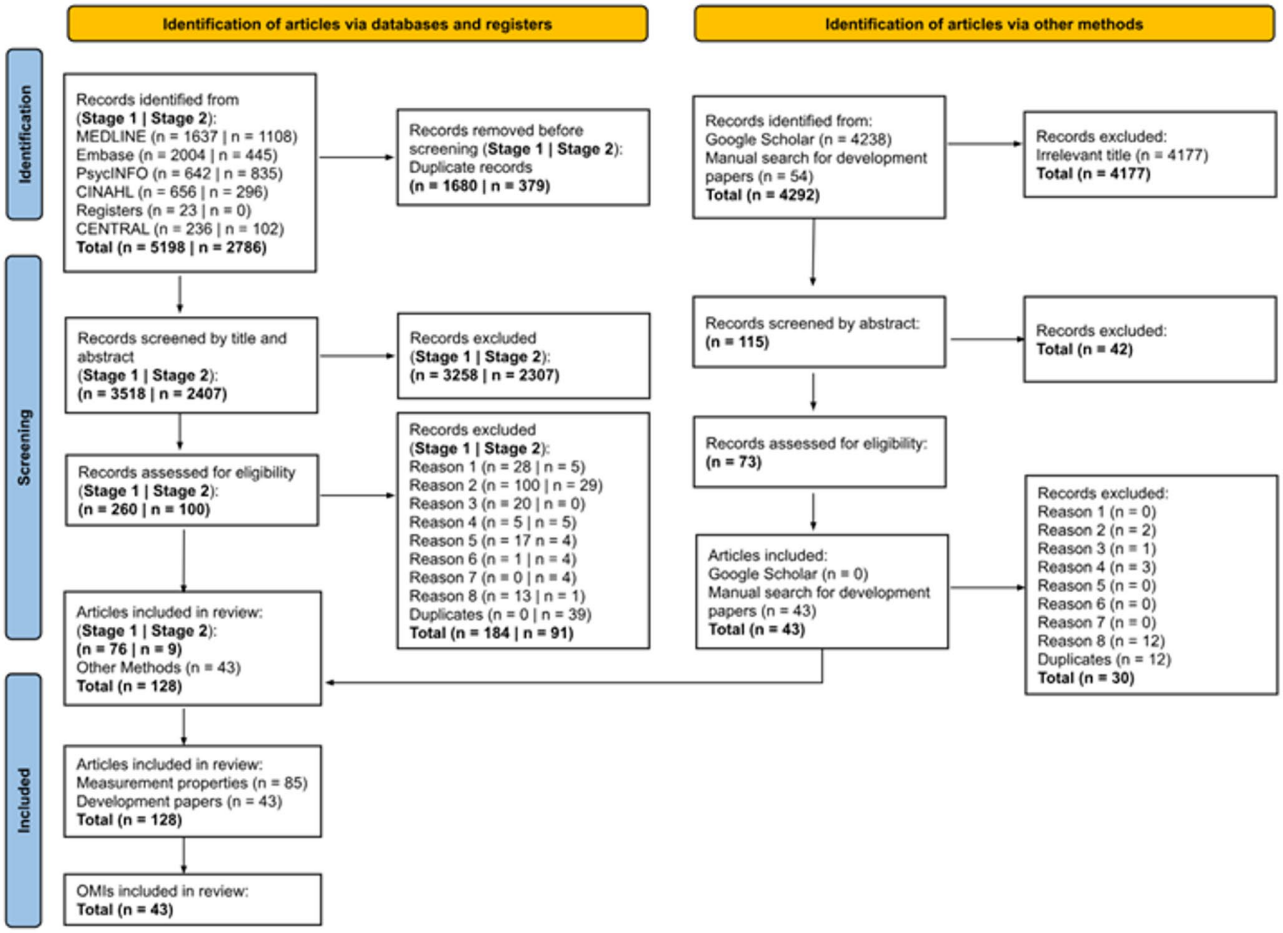
PRISMA-COSMIN Diagram Flowchart adapted according to PRISMA-COSMIN template [26] for Search 1, pertaining to full texts meeting eligibility criteria, and Search 2, pertaining to articles filtered for measurement properties.Reasons for record exclusions: (1) Title and abstract not written in English in a peer-reviewed journal. (2) Not a primary research article with full-text available. (3) Participants are not adult informal carers ≥ 18. (4) Participants are not informal carers for individuals with ALS. (5) HRQoL, or domain/s of HRQoL are not assessed by a freely available, multi-item outcome measurement tool. (6) Articles with mixed syndromic groups have < 75% ALS informal carers, or separate data is not available for ALS informal carers. (7) Not a development article of a HRQoL measure used with ALS informal carers, does not report data on the content validity of HRQoL measures, does not use HRQoL measures with ALS carers and report HRQoL scores. (8) HRQoL PROM development article could not be sourced or unavailable in full text in English. CENTRAL = Cochrane Central Register of Controlled Trials. CINAHL = Cumulative Index of Nursing and Allied Health Literature. COSMIN = Consensus-based Standards for the Selection of Health Measurement Instruments. HRQoL = Health-related quality of life. MEDLINE = Medical Literature Analysis and Retrieval System Online. OMI = Outcome Measurement Instrument. PRISMA = Preferred Reporting Items for Systematic Reviews and Meta-Analyses. PROM = Person Reported Outcome Measure

Multi-item, freely available, self-report PROMs or PROM subscales were eligible for inclusion if they measured a minimum of one component of HRQoL in adult informal carers of people living with ALS. Single-item PROMs, such as the EuroQoL Visual Analogue Scale [16]were not eligible for inclusion as psychometric standards require more than one item to permit rigorous evaluation [22, 23]. Copies of the HRQoL PROMs identified from Search 1 were independently screened by two researchers (RB and JC) to determine whether the PROM met predetermined eligibility criteria. This involved consideration of PROM content to determine whether aforementioned eligibility criteria was met. Where disagreement occurred, a third researcher (TS) ratified the inclusion decision. Reasons for PROM and PROM development article exclusion are listed in PRISMA diagram (Fig. 1).

### Assessment of PROM development articles

Data extraction tools were developed according to COSMIN reporting guidelines [35] (see Supplementary Material 2). Data extraction was completed by one reviewer (RB). Methodological quality of included PROM development articles was independently assessed by two reviewers (RB and JC) with consensus reached via discussion. To rate methodological quality, each COSMIN standard (or item) was measured using a four-point scale from ‘*Inadequate’*,* ‘Doubtful’*,* ‘Adequate’ to ‘Very Good*’ [36]. Consistent with COSMIN methods [37]the final rating across COSMIN standards for each article was determined by the lowest rating assigned to any standard. For example, if any aspect of ‘PROM design’ was rated ‘*Inadequate*’, this yields an overall rating of ‘*Inadequate*’ despite presence of ‘*Very Good*’ ratings for other standards.

### Assessment of content validity

The assessment of content validity for each PROM involves evaluation of evidence from three sources: (1) the quality of the PROM development article; (2) the quality of PROM content validity articles; and (3) evaluation of PROM content by the research team. Relevance, comprehensiveness and comprehensibility ratings were made for each source of evidence independently by two researchers (RB and JC) with consensus reached via discussion. Individual ratings for content validity, and its constituent components of relevance, comprehensibility and comprehensiveness, were qualitatively synthesised using COSMIN rating synthesis rules [22] (Sheet 7, Supplementary Material 3). Using these rules, each PROM could receive an overall synthesised rating of ‘*Sufficient*’ (+), ‘*Inconsistent*’ (±) or ‘*Insufficient*’ (-). For example, if the PROM development article was rated ‘*Insufficient*’ (-) and the researcher rating was ‘*Inconsistent*’ (±) for comprehensibility, the overall synthesised comprehensibility rating would be ‘*Insufficient*’ (-). In the first instance, COSMIN rating synthesis rules [22] were used to combine scores for relevance, comprehensiveness and comprehensibility. When aforementioned synthesis rules could not be applied to scores, rating synthesis rules from previous COSMIN reviews [31, 38] were utilised (Supplementary Material 3).

Quality of evidence was independently rated by two researchers (RB and JC) using the COSMIN-modified GRADE approach [23] and rated as ‘*High*’, ‘*Moderate*’, ‘*Low*’ or ‘*Very Low*’. Evidence was initially rated as high quality, then downgraded according to four components: (1) risk of bias, (2) inconsistency, (3) imprecision and (4) indirectness [22]. ‘*Low*’ quality rating equates to high risk of bias, whilst a rating of ‘*High*’ equates to low risk of bias. Risk of bias, content validity and certainty assessments were considered when formulating recommendations for which PROM or PROM subscale, if any, were best suited to assessing HRQoL of informal carers of people living with ALS, considering current available evidence.

## Results

### Article selection

Search 1 generated 5198 records (Fig. 1). After duplicates were removed, 3518 records were screened via title and abstract. A total of 260 records were assessed for eligibility via full-text. 184 were rejected and 76 articles were included in this review. In Search 2, 2786 records were identified, from which 379 duplicates were removed and 2407 were screened via title and abstract. One hundred records were assessed for eligibility with nine articles eligible for inclusion. Cohen’s kappa of inter-rater reliability for full-text review for Search 1 and 2 was κ = 0.65, interpreted as ‘*substantial agreement’* [39]. Additionally, 4292 records were screened from other sources (i.e., Google Scholar searches and manual searching for development articles). From these sources, 73 records were eligible for full text review, and 43 development articles were ultimately included. No new articles were found from Google Scholar that were not already identified in database searches. Overall, of the 12,276 records reviewed, 12,148 were rejected and 128 were accepted for inclusion in this review (85 articles providing evidence of measurement properties and 43 PROM development articles). A complete reference list of included articles is available in Supplementary Material 4.

### PROMs identified for review

From the 76 eligible full-texts from Search 1, 109 distinct PROMs were used with adult informal carers in ALS and 43 were eligible for inclusion (Table 2). Supplementary Material 5 contains the full list of 109 PROMs with reasons for exclusion. The number of PROMs used per article ranged from 1 to 11 with a median of 2 (IQR = 2–3). Individual PROMs were used in a total of 1–30 articles, with a median of 1 (IQR = 1–2). Of the included PROMs, the Zarit Burden Interview (ZBI) was used across the highest number of articles (*n* = 30), followed by the Hospital Anxiety and Depression Scale (HADS) (*n* = 24), Carer Burden Inventory (CBI) and Carer Strain Index (CSI) (*n* = 10). Supplementary Material 6 details full information on the frequency of PROM use in the included articles.

**Table 2:** Summary of PROMs and PROM subscales from stage 1 search *Aspects of HRQoL as defined by PROM developer in PROM development article. HRQoL = Health-Related quality of life. QoL = quality of life

PROM or PROM Subscale	Recall period	*N* subscales (*N* items)	Total score (Y/*N*)	HRQoL domains assessed*	Response option type (*N* options)	Origin language (Country)	Target population
Acceptance of Illness Scale (AIS)	N/A	0 (8)	Y	Psychological acceptance	Frequency (5)	English (US)	Adult patients with chronic illness - non-hospitalised
Beck Depression Inventory (BDI)	Present	0 (21)	Y	Depression	Varies (4–5)	English (US)	Adult patients with suspected symptoms of depression
Beck Hopelessness Scale (BHS)	Present	0 (20)	Y	Hopelessness	True/ False	English (UK)	Adult patients
Coping Orientation to Problems Experienced Inventory (Brief COPE)	N/A	0 (28)	Y	Coping	Frequency (4)	English (US)	General adult population
Burden Scale for Family Caregivers (BSFC)	N/A	0 (28)	Y	Caregiver Burden	Agreement (4)	German (Germany)	Adult carers
Carer Quality of Life (CarerQoL)	Present	7 (7 + VAS)	Y	Caregiver Burden	Severity (4)	Dutch (Netherlands) (assumed)	Informal adult caregivers
Caregiver Burden Inventory (CBI)	N/A	5 (24)	Y	Caregiver Burden	Frequency (5)	English (US)	Adult carers
Center for Epidemiology Articles Depression Scale (CES-D-10)	Past week	0 (20)	Y	Depression	Frequency (4)	English (US)	General adult population
Chalder Fatigue Scale (CFS)	N/A	2 (14)	Y	Physical and Mental Fatigue	Severity (4)	English (UK)	General adult population
Chalder Fatigue Scale - Physical Fatigue Subscale (CFS-Physical	N/A	0 (8)	Y	Physical Fatigue	Severity (4)	English (UK)	General adult population
Caregiver Network Scale (CNS)	Present	4 (50)	Y	Caregiver Social Support	Agreement (5)	English (Australia)	Adult carers of people living with ALS
Close Persons Questionnaire (CPQ)	N/A	3 (15)	Y	Social Support	Frequency (5)	English (UK)	General adult population
Caregiver Strain Index (CSI)	N/A	0 (13)	Y	Caregiver strain	Agreement (2)	English (US)	Adult carers
Dyadic Adjustment Scale - Dyadic Subscale (DAS)	N/A	5 (32)	Y	Quality of Dyadic Relationship	Varies by item (varies by item)	English (US)	General adult population
EQ-5D-5L	Today	5 (5 + VAS)	Y	Health status	Severity (5)	English (UK) and Spanish	General adult population
Existential Well-Being Subscale from the McGill Quality of Life Questionnaire (EWBS)	Last 2 Days	0 (3)	Y	Meaningful Existence	Agreement (10)	English (US)	Adult patients
Functional Assessment of Chronic Illness Therapy–Spiritual Well-Being Scale (FACIT-Sp)	Past 7 Days	2 (12)	Y	Spiritual Wellbeing	Agreement (5)	English (US)	Adult patients
The Duke-UNC Functional Social Support Questionnaire (FSSQ)	N/A	2 (8)	Y	Social Support	Frequency (5)	English (US)	General adult population
General Health Questionnaire (GHQ)	Recently	4 (28)	Y	Psychological symptoms	Severity (4)	English (UK)	Adult patients
Hospital Anxiety & Depression Scale (HADS)	Last week	2 (14)	N	Anxiety, depression	Frequency (4)	English (UK)	Adult patients
Life Satisfaction Checklist (LiSat-11)	N/A	0 (11)	Y	Happiness - Life Satisfaction	Satisfaction (6)	Swedish (Sweden)	General adult population
Metacognitive Questionnaire 30 (MCQ-30)	N/A	5 (30)	Y	Metacognition	Agreement (4)	English (UK)	Adult patients
Multidimensional Scale of Perceived Social Support (MPSS)	N/A	3 (12)	Y	Social Support	Agreement (7)	English (US)	General adult population
McGill Quality of Life Questionnaire (MQOL)	Last 2 Days	4 (17)	Y	QoL	Agreement (10)	English (US)	Adult patients
Positive and Negative Affect Schedule (PANAS)	N/A	2 (20)	Y	Affect	Severity (5)	English (US)	General adult population
Patient Health Questionnaire-9 (PHQ-9)	Last 2 Weeks	0 (9)	Y	Depression	Frequency (4)	English (US)	Adult patients
Profile of Mood States - Short Form (POMS-SF)	N/A	6 (37)	Y	Psychological Distress	Severity (5)	English (US)	Adults - patients and general population
QoL Enjoyment & Satisfaction Questionnaire Short Form (Q-LES-Q-SF)	Past week	0 (16)	Y	QoL	Satisfaction (5)	English (US)	Adult patients
Quality of Life in Life-Threatening Illness Family Carer Version (QOLLTI-F)	Past 2 days	0 (16)	Y	QoL	Agreement (11)	English (US)	Adult carers
Quality of Life at the End of Life (QUAL-E-Fam)	Last week	2 (17)	Y	QoL	Severity (5) and Frequency (5)	English (US)	Adult carers
Rand 36-Item Health Survey (RAND-36)	Past 4 Weeks	8 (36)	Y	HRQoL	Varies (2–6)	English (US)	General adult population
Self-Rating Anxiety Scale (SAS)	Past several days	0 (20)	Y	Anxiety	Frequency (4)	English (US)	Adult patients
Self-Rating Depression Scale (SDS)	Past several days	0 (20)	Y	Depression	Frequency (4)	English (US)	Adult patients
Short Form-12 (SF-12)	Varies by item	2 or 8 (12)	N	HRQoL	Varies by item (varies by item)	English (US)	General adult population
Short Form-36 (SF-36)	Varies by item	2 or 8 (36)	N	HRQoL	Varies by item (varies by item)	English (US)	Adult patients and general population
Short Form-36 Mental Component Summary (SF-36 MCS)	Varies by item	4 (14)	N	HRQoL	Varies by item (varies by item)	English (US)	Adult patients and general population
Short Form-36 Version 2 (SF-36 V2)	Varies by item	2 or 8 (36)	N	HRQoL	Varies by item (varies by item)	English (US)	Adult patients and general population
State-Trait Anxiety Inventory-X (STAI-X)	Present (state); General (trait)	2 (40)	Y	State & Trait Anxiety	Severity (state) (4); Frequency (trait) (4)	English (US)	General adult population
State-Trait Anxiety Inventory-Y1 (STAI-Y1)	Present	1 (20)	Y	State & Trait Anxiety	Severity (state) (4); Frequency (trait) (4)	English (US)	General adult population
State-Trait Anxiety Inventory-Y (STAI-Y1 & Y2)	Present and general	2 (40)	Y	State & Trait Anxiety	Severity (state) (4); Frequency (trait) (4)	English (US)	General adult population
Satisfaction With Life Scale (SWLS)	N/A	0 (5)	Y	Life satisfaction	Agreement (3)	English (UK)	Unclear
World health organisation quality of life-BREF (WHOQOL-BREF)	2 weeks	4 (26)	Y	QoL	Varies by item (4)	Multiple	Adult patients, carers, and general population
Zarit Burden Interview (ZBI)	N/A	0 (22)	Y	Caregiver burden	Frequency (5)	English (US)	Adult carers

### Assessment of PROM development articles

Ratings for PROM development articles for ten PROMs were extracted from a prior review [38] (Beck Depression Inventory [BDI], Caregiver Strain Index [CSI], Carer Quality of Life [CarerQoL], EQ-5D-5 L, Hospital Anxiety and Depression Scale [HADS], Short Form-12 [SF-12], Short Form-36 [SF-36], State Trait Anxiety Inventory-X [STAI-X], World Health Organisation Quality of Life-BREF [WHOQOL-BREF] and Zarit Burden Interview [ZBI]). Two development articles were used to assess four PROMs (Supplementary Material 4). PROM development articles produced ‘*Inadequate*’ ratings for all but three PROMs: the Close Persons Questionnaire (CPQ), EQ-5D-5L and Quality of Life at the End of Life–Family Carer Version (QUAL-E-Fam). These PROMs were rated as ‘*Doubtful*’ and surpassed an ‘*Inadequate*’ rating due to the presence of cognitive interview methods within PROM development and simultaneously were not scored down for other factors within PROM design or development.

### Assessment of content validity

No articles reporting on the content validity of HRQoL PROMs used with informal carers in ALS were identified. Therefore, the assessment of content validity was conducted by combining evidence from the PROM development papers and reviewer ratings, consistent with COSMIN methods for synthesising ratings [22] (Table 3). Overall ratings for relevance, comprehensiveness and comprehensibility were combined to produce ‘*Inconsistent*’ or ‘*Insufficient*’ overall content validity ratings for all 43 PROMs. Supplementary Material 3 contains COSMIN rating sheets. The CarerQoL [18] was the only PROM to achieve an ‘*Inconsistent*’ overall rating for comprehensiveness within this review.

**Fig. 2:**
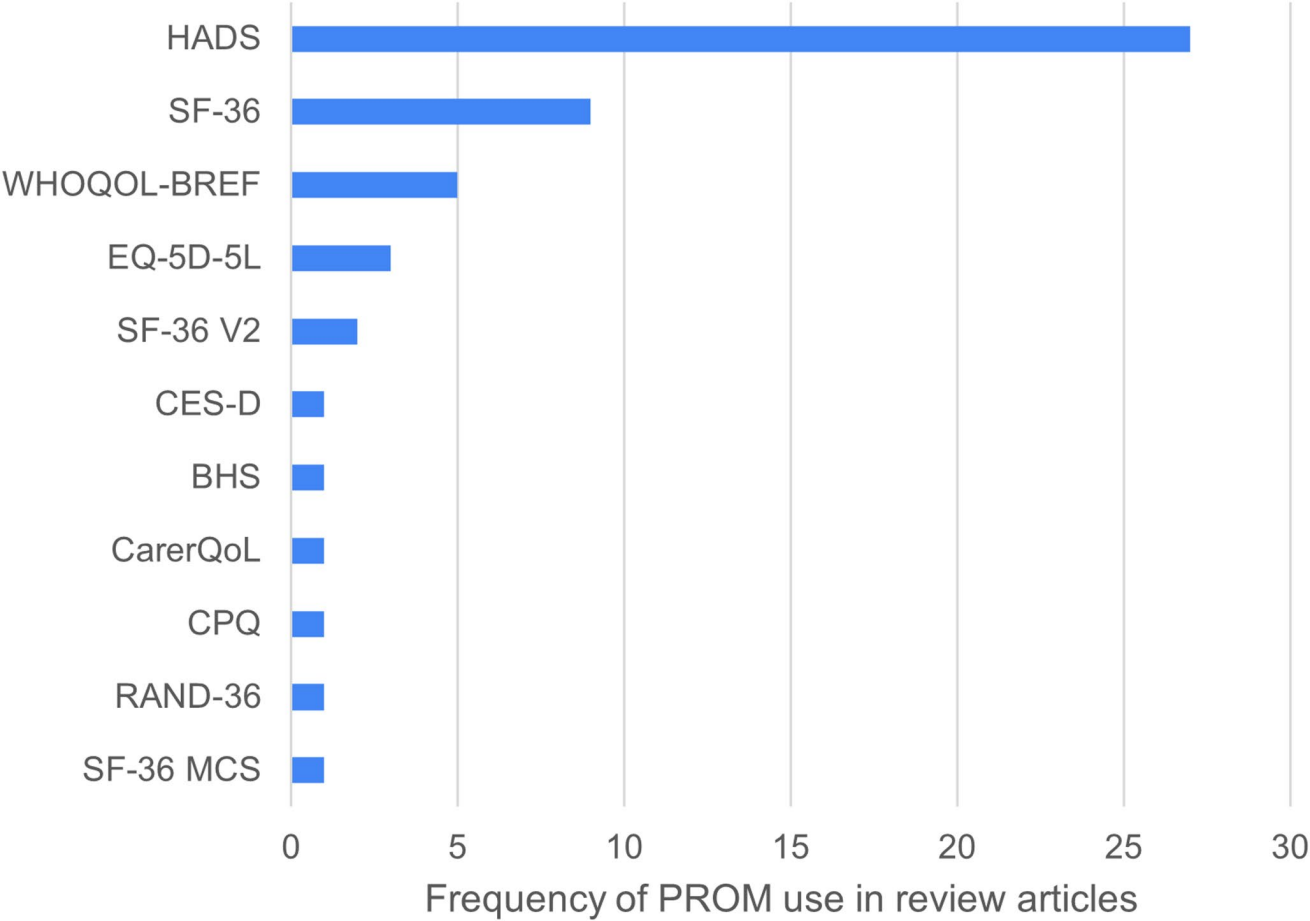
Frequency diagram illustrating PROMs with highest overall content validity and highest frequency of use within articles in this review. HADS = Hospital Anxiety & Depression Scale, RAND-36 = Rand 36-Item Health Survey, SF-36 = Short Form-36, SF-36 MCS = Short Form-36 Mental Component Summary, SF-36 V2 = Short Form-36 Version 2, WHOQOL-BREF = World health organisation quality of life-BREF

The 11 PROMs receiving an ‘*Insufficient*’ rating for overall content validity were all rated ‘*Inadequate*’ for their respective development study. Those PROMs with an ‘*Inconsistent*’ rating either had better ratings for their respective development study or were rated more favourably in reviewer ratings (Supplementary Material 3). PROMs with highest overall content validity ratings and highest frequency of use are shown in Fig. 2.

Quality of evidence supporting content validity ratings was ‘*Very Low*’ for all PROMs with the exception of the CPQ, EQ-5D-5L and QUAL-E (Fam). Quality assessment scores are shown in Table 3. Consistent with the COSMIN-modified GRADE approach [22]all PROMs began with a ‘*Moderate*’ rating due to the lack of content validity articles with ALS carers. This baseline rating was adjusted based on the quality of evidence in PROM development articles. Quality ratings could have been universally downgraded to ‘*Very Low*’ due to ‘*Indirectness*’, as all PROMs within this review, with the exception of the CNS, were not developed with informal carers or people with ALS. PROMs were not downgraded further to ensure quality assessment could distinguish between PROMs based on the varying quality of their development articles.

**Table 3:** Content validity ratings across all 43 PROMs and PROM subscales considering quality of evidence from development articles, reviewer ratings and overall synthesised content validity ratings. Content validity ratings are broken down into relevance, comprehensiveness and comprehensibility for aforementioned sections. Ratings for the quality of development articles are on a 4-point scale: I = ‘Inadequate’, D = ‘Doubtful’, A = ‘Adequate’ to V = ‘Very good’. Ratings for relevance, comprehensibility and comprehensiveness are on a 4-point scale: (+) sufficient, (±) inconsistent, (?) indeterminate, and (–) insufficient. Ratings for overall content validity is via a 3-point scale: (+) sufficient, (±) inconsistent, and (–) insufficient

PROM or PROM Subscale		Development Article		Overall Ratings				Overall Content Validity	Quality of Evidence
		COSMIN Quality Rating	Were Carers Involved?		Relevance	Comprehensiveness	Comprehensibility		
Acceptance of Illness Scale	AIS	I	N	±		-	-	-	Very Low
Beck Depression Inventory	BDI	I	N	-		-	+	±	Very Low
Beck Hopelessness Scale	BHS	I	N	±		-	+	±	Very Low
Coping Orientation to Problems Experienced Inventory	Brief COPE	I	N	±		-	±	±	Very Low
Burden Scale for Family Caregivers	BSFC	I	Y	-		-	±	-	Very Low
Carer Quality of Life	CarerQoL	I	Y	±		±	+	±	Very Low
Caregiver Burden Inventory	CBI	I	Y	±		-	±	±	Very Low
Center for Epidemiology Articles Depression Scale	CES-D-10	I	N	±		-	+	±	Very Low
Chalder Fatigue Scale	CFS	I	N	±		-	-	-	Very Low
Chalder Fatigue Scale - Physical Fatigue Subscale	CFS-Physical	I	N	±		-	-	-	Very Low
Caregiver Network Scale	CNS	I	Y	±		-	±	±	Very Low
Close Persons Questionnaire	CPQ	D	N	±		-	+	±	Low
Caregiver Strain Index	CSI	I	Y	±		-	±	±	Very Low
Dyadic Adjustment Scale - Dyadic Subscale	DAS	I	N	±		-	-	-	Very Low
EuroQoL-5 Dimensions	EQ-5D-5 L	D	N	±		-	+	±	Low
Existential Well-Being Subscale from the McGill Quality of Life Questionnaire	EWBS	I	N	-		-	±	-	Very Low
Functional Assessment of Chronic Illness Therapy–Spiritual Well-Being Scale	FACIT-Sp	I	N	±		-	-	-	Very Low
The Duke-UNC Functional Social Support Questionnaire	FSSQ	I	N	±		-	±	±	Very Low
General Health Questionnaire	GHQ	I	N	±		-	-	-	Very Low
Hospital Anxiety & Depression Scale	HADS	I	N	±		-	+	±	Very Low
Life Satisfaction Checklist	LiSat-11	I	N	-		-	±	-	Very Low
Metacognitive Questionnaire 30	MCQ-30	I	N	±		-	±	±	Very Low
Multidimensional Scale of Perceived Social Support	MPSS	I	N	±		-	±	±	Very Low
McGill Quality of Life Questionnaire	MQOL	I	N	-		-	±	-	Very Low
Positive and Negative Affect Schedule	PANAS	I	N	±		-	±	±	Very Low
Patient Health Questionnaire-9	PHQ-9	I	N	±		-	±	±	Very Low
Profile of Mood States - Short Form	POMS-SF	I	N	±		-	±	±	Very Low
QoL Enjoyment & Satisfaction Questionnaire Short Form	Q-LES-Q-SF	I	N	±		-	±	±	Very Low
Quality of Life in Life-Threatening Illness Family Carer Version	QOLLTI-F	I	Y	-		-	-	-	Very Low
Quality of Life at the End of Life	QUAL-E (fam)	D	Y	±		-	±	±	Low
Rand 36-Item Health Survey	RAND-36	I	N	+		-	+	±	Very Low
Self-Rating Anxiety Scale	SAS	I	N	±		-	+	±	Very Low
Self-Rating Depression Scale	SDS	I	N	±		-	+	±	Very Low
Short Form-12	SF-12	I	N	±		-	+	±	Very Low
Short Form-36	SF-36	I	N	+		-	+	±	Very Low
Short Form-36 Mental Component Summary	SF-36 MCS	I	N	+		-	+	±	Very Low
Short Form-36 Version 2	SF-36 V2	I	N	+		-	+	±	Very Low
State-Trait Anxiety Inventory-X	STAI-X	I	N	±		-	+	±	Very Low
State-Trait Anxiety Inventory-Y1	STAI-Y1	I	N	±		-	±	±	Very Low
State-Trait Anxiety Inventory-Y	STAI-Y1 & Y2	I	N	±		-	±	±	Very Low
Satisfaction With Life Scale	SWLS	I	N	-		-	+	±	Very Low
World health organisation quality of life-BREF	WHOQOL-BREF	I	N	+		-	+	±	Very Low
Zarit Burden Interview	ZBI	I	Y	-		-	+	±	Very Low

## Discussion

This review is the first of its kind to systematically assess the content validity of PROMs (or PROM subscales) used to measure HRQoL (or a component thereof) in adult informal carers of people with ALS, using current best practice guidance. We identified a wide range of PROMs used for this purpose. The number of PROMs used per article varied (ranging from 1 to 11), with the Zarit Burden Interview (ZBI) used most frequently across all articles. Our results revealed a lack of evidence supporting the content validity of identified PROMs, questioning their ability to fully capture the impact of caregiving on the HRQoL of informal carers in ALS.

Informal carers’ HRQoL outcomes have been shown to be inextricably linked [40–42] with those of their care recipient in ALS and therefore should be a central consideration for clinical decision-making. A concordance exists in the outcomes of depression and distress between informal carers and people living with ALS [40–42]. Carer distress has been shown to negatively impact quality of care for people with ALS and their ability to remain at home to receive their care [7]. Further, living without an informal carer has been identified as an independent predictor of reduced prognosis in ALS [43]. The lack of evidence for the content validity of PROMs identified in this review, means the current literature on the impact of being an informal carer is limited (if not flawed) and needs to be interpreted with caution.

The absence of robust qualitative methods in developing HRQoL PROMs is an important factor contributing to the results of this review. The prevalence of ‘*Inadequate*’ ratings for PROM development articles was influenced by the limited inclusion of informal carers within qualitative PROM development methods and does not necessarily mean a PROM is not fit for purpose. Modern approaches to PROM development favours consultation with individuals with lived experience of a particular phenomenon (i.e., informal caregiving in ALS), known as ‘bottom-up’ methodology [44, 45]. In contrast, historical ‘top-down’ methods [46] rely on research literature or expert consultation with clinicians or academics. Only eight PROMs within this review were designed specifically for informal carers. Two of which utilised ‘bottom-up’ qualitative methods with informal carers to inform PROM development: the Quality of Life at the End of Life (QUAL-E-Fam) utilised qualitative interview methods to generate and refine PROM items and the Quality of Life in Life-Threatening Illness Family Carer Version (QOLLTI-F) consulted informal carers to review the comprehensibility and acceptability of items initially generated via top-down methods. Of PROMs included within this review, the Caregiver Network Scale (CNS) was the only PROM developed specifically for informal carers in ALS. Nonetheless, this was derived from top-down methods via a literature review and expert consultation.

Given the contemporary shift towards bottom-up PROM development methods, it is unrealistic to expect older PROMs (termed ‘legacy measures’) to have used these methods, as their formation predated current international PROM development standards. However, the absence of bottom-up methods in legacy measure development does not necessarily indicate their invalidity. For instance, the ZBI, developed in 1960, received an ‘*Inconsistent*’ rating for overall content validity but was the most frequently used PROM in this review. Understanding why legacy measures, like the ZBI, are prevalent in ALS carer literature is important, yet most articles in this review do not explain their choice of PROMs. The ZBI’s frequent use could be justified by its extensive language validation [47] and could also imply acceptability and validity amongst respondents, although frequency of use cannot serve as a proxy measure of either of these concepts. Future studies should provide justification for their choice of PROMs in their reporting to improve the quality of HRQoL literature and ensure the use of appropriate PROMs for their specific construct, context and target population.

Whilst the ZBI’s frequent use in this review could infer acceptability for informal carers in ALS, the concept of acceptability remains subjective, complex and challenging to define and assess. Current definitions of acceptability vary but include considerations such as suitability, convenience and effectiveness of a PROM for a target population [48]. COSMIN methods do not include consideration of acceptability. Whilst a PROM may receive a favourable content validity rating, this does not necessarily infer favourable acceptability for respondents. For example, a highly comprehensive PROM which includes many items may not be acceptable due to respondent burden. Conversely, a brief PROM with few items may be acceptable in terms of respondent burden but be inadequate in terms of comprehensibility. Further qualitative research with informal carers in ALS is required to establish the acceptability of HRQoL PROMs that are otherwise deemed psychometrically appropriate for this target population and construct.

In the absence of further research with informal carers in ALS, this review recommends considering the CarerQoL for measuring HRQoL in this cohort. This recommendation is based on the superior comprehensiveness rating for the CarerQoL, indicating that this PROM currently provides the best available evidence for encompassing the psychological, physical and social aspects of caregiving amongst PROMs included in this review. Use of one comprehensive HRQoL PROM could negate use of multiple PROMs for informal carers, reducing repetition of concepts or items across multiple PROMs and minimising the cognitive load associated with switching between response options and recall periods. Nevertheless, it is worth noting that whilst the CarerQoL was rated favourably via COSMIN methods, it was not frequently used amongst articles within this review.

This review is not without its limitations. Despite following current best practice, there have been criticisms of COSMIN methodology. These include the retrospective nature of COSMIN evaluation, thought to unfavourably rate newly developed PROMs [49]and its potential unsuitability for assessing legacy measures. Firstly, emphasis on transparency of methodological reporting has increased over time. Within this review, median publication date of PROM development articles was 1992 (IQR = 1985–1997), highlighting the predominance of legacy measures, which were rated poorly due to the lack of reporting transparency and predominance of top-down development methods. Crucially, this review identified no content validity articles for any of the HRQoL PROMs which could provide evidence to support their use. There is a need for content validity articles for frequently used legacy measures to support (or question) their ongoing use.

Secondly, within COSMIN methodology, final ratings are determined by applying the lowest rating for any item within an article [37]. These methods can be considered harsh, especially when rating legacy measures, and have the potential to bias higher ratings to more recently developed PROMs with greater methodological reporting transparency. Thirdly, COSMIN guidance for synthesising content validity ratings were not applicable for almost half of the PROMs within this review. These PROMs produced combinations of ratings for relevance, comprehensiveness and comprehensibility that could not be applied to rating synthesis rules published within COSMIN methods [22]. In these cases, additional synthesis rules from prior COSMIN reviews [31, 38] were used to combine ratings (Supplementary Material 3).

Finally, despite a robust search strategy using validated filters [29, 30]other PROMs, or articles documenting the psychometric properties of PROMs, for informal carers in ALS may exist. Restricting searches to English may have excluded development papers or PROMs from non-Anglophone settings, potentially biasing findings. This review excluded articles with mixed carer cohorts if ALS carers constituted less than 75%, potentially omitting relevant data from smaller cohorts. Additionally, some identified PROMs (*n* = 23) could not be sourced (Supplementary Material 4), although their limited availability suggests they are not widely used in clinical practice. Furthermore, there may be PROMs developed for carers in other health conditions (that have not currently been described in published research), which may be valid and acceptable for ALS carers.

The striking paucity of evidence for the content validity of HRQoL PROMs for informal carers in ALS is of substantial concern for both research and clinical practice. Existing PROM development studies are of low quality and there is a lack of evidence supporting PROM content validity for this cohort. Current literature reporting the impact of informal caregiving in ALS is hence inherently limited, as existing HRQoL PROMs may underestimate or overlook critical physical, psychological or social impacts of caregiving in ALS. Further, many HRQoL PROMs in use are ‘legacy measures’ that may no longer reflect the complexities of modern caregiving. There is an urgent need for high-quality research to assess the validity and acceptability of existing HRQoL PROMs for informal carers in ALS to support and guide clinical decision-making for this cohort. This review has highlighted key evidence gaps in PROMs currently available to quantify the impact of informal caregiving on HRQoL in the context of ALS. Accordingly, future research is needed to address two areas. Firstly, the generation of evidence to support the content validity of existing PROMs for use within ALS carers, including consideration of acceptability for the target population. Secondly, to investigate the psychometric performance of those PROMs which are found to be acceptable. The use of unsupported PROMs in clinical practice risks underestimating the true impact of caregiving, which is a vital consideration given the concordance between carer and care recipient HRQoL outcomes in ALS.

## Electronic supplementary material

Below is the link to the electronic supplementary material.

Supplementary Material 1

Supplementary Material 2

Supplementary Material 3

Supplementary Material 4

Supplementary Material 5

Supplementary Material 6

Supplementary Material 7

## Glossary

ALS: Amyotrophic Lateral Sclerosis
COSMIN: Consensus-based Standards for the Selection of Health Measurement Instruments
FTD: Frontotemporal Dementia
HRQoL: Health Related Quality of Life
PRISMA: Preferred Reporting Items for Systematic Reviews and Meta-Analyses
PROMs: Person Reported Outcome Measures
PROSPERO: International Prospective Register of Systematic Reviews
QoL: Quality of Life
