# Supplementary material for: Health-related quality of life of informal carers in ALS: a systematic review of person reported outcome measures
Source: Qual Life Res. 2025 Jun 25;34(10):2731–44. doi: 10.1007/s11136-025-04012-y (PMC12535500; doi:10.1007/s11136-025-04012-y)
Supplement: Supplementary file 1 — Supplementary Material 1 [file 11136_2025_4012_MOESM1_ESM.docx]

**Supplementary Material 1: Search Strategy, Yield and List of Databases**

**Title:** Health-Related Quality of Life of Informal Carers in ALS: A Systematic Review of Person Reported Outcome Measures

**Journal:** Quality of Life Research

**Authors:** Ms Rosie Bamber, Dr Theocharis Stavroulakis, Professor Christopher McDermott and Professor Jill Carlton

**Corresponding Author:**

Professor Jill Carlton, PhD

Professor of Health Outcomes

Sheffield Centre for Health and Related Research (SCHARR)

University of Sheffield

j.carlton@sheffield.ac.uk

**Stage 1 Search**

| **Host** | **Database** | **Dates Covered** | **Date Searched** | **Search Yield** |
| --- | --- | --- | --- | --- |
| Ovid | MEDLINE(R) and Epub Ahead of Print, In-Process & Other Non-Indexed Citations, Daily and Versions(R) | 1946 - Date | 24/11/2023 | 1637 |
| Ovid | Embase | 1974 - Date | 24/11/2023 | 2004 |
| Ovid | PsycINFO | 1806 - Date | 24/11/2023 | 642 |
| EBSCO | CINAHL | 1974 - Date | 24/11/2023 | 656 |
| Wiley | Cochrane Database of Systematic Reviews | 1996 - Date | 24/11/2023 | 23 |
| Wiley | Cochrane Central Register of Controlled Trials | 1898 - Date | 24/11/2023 | 236 |
| **TOTAL** | | | | **5198** |

No restrictions to the publication date were applied to articles. Databases were searched separately with specific search criteria:

PROMS filter source:

<https://cosmin.nl/wp-content/uploads/prom-search-filter-oxford-2010.pdf> [Accessed on: 24/11/2023]

**Ovid MEDLINE(R) and Epub Ahead of Print, In-Process & Other Non-Indexed Citations, Daily and Versions(R)**

**1946 to 23rd November 2023**

24th November 2023

| **#** | **Search Terms** | **Results** |
| --- | --- | --- |
| 1 | (HR-PRO or HRPRO or HRQL or HRQoL or QL or QoL).ti,ab. or quality of life.mp. or (health index* or health indices or health profile*).ti,ab. or health status.mp. or ((patient or self or child or parent or carer or proxy) adj (appraisal* or appraised or report or reported or reporting or rated or rating* or based or assessed or assessment*)).ti,ab. or ((disability or function or functional or functions or subjective or utility or utilities or wellbeing or well being) adj2 (index or indices or instrument or instruments or measure or measures or questionnaire* or profile or profiles or scale or scales or score or scores or status or survey or surveys)).ti,ab. | 999,514 |
| 2 | exp Caregivers/ or carer*.mp. or caring.mp. or carergiv*.mp. or care-giv*.mp. or spillover*.mp. or spill over*.mp. or partner*.mp. or husband.mp. or wife.mp. or spouse*.mp. or spousal.mp. or child.mp. or children.mp. or son.mp. or sons.mp. or daughter*.mp. or offspring*.mp. or parent*.mp. or mother*.mp. or maternal.mp. or father*.mp. or paternal.mp. or sibling*.mp. or brother*.mp. or sister*.mp. or dependent*.mp. or "next of kin".mp. or kinship.mp. or grandparent*.mp. or grandmother*.mp. or grandfather*.mp. or relative*.mp. or family.mp. or "close person*".mp. or families.mp. | 7,609,943 |
| 3 | (ALS or "Amyotrophic lateral sclerosis").mp. or exp Motor Neuron Disease/ or Motor neuron* disease*.mp. or Motor neuron disease*.mp. or MND.mp. or Charcot* disease.mp. or "Charcot* disease".mp. or anterior horn cell disease*.mp. or Lateral sclerosis.mp. or motor system disease*.mp. or primary lateral sclerosis.mp. or PLS.mp. or progressive muscular atrophy.mp. or PMA.mp. or progressive bulbar palsy.mp. or PBP.mp. or "Lou Gehrig* disease".mp. | 140,911 |
| 4 | #1 AND #2 AND #3 | 1637 |

**Embase via Ovid**

**1946 to 23rd November 2023**

24th November 2023

| **#** | **Search Terms** | **Results** |
| --- | --- | --- |
| 1 | (HR-PRO or HRPRO or HRQL or HRQoL or QL or QoL).ti,ab. or quality of life.mp. or (health index* or health indices or health profile*).ti,ab. or health status.mp. or ((patient or self or child or parent or carer or proxy) adj (appraisal* or appraised or report or reported or reporting or rated or rating* or based or assessed or assessment*)).ti,ab. or ((disability or function or functional or functions or subjective or utility or utilities or wellbeing or well being) adj2 (index or indices or instrument or instruments or measure or measures or questionnaire* or profile or profiles or scale or scales or score or scores or status or survey or surveys)).ti,ab. | 1,514,465 |
| 2 | (carer* or caring or carergiv* or care-giv* or spillover* or spill over* or partner* or husband or wife or spouse* or spousal or child or children or son or sons or daughter* or offspring* or parent* or mother* or maternal or father* or paternal or sibling* or brother* or sister* or dependent* or "next of kin" or kinship or grandparent* or grandmother* or grandfather* or relative* or family or "close person*" or families).mp. | 9,407,977 |
| 3 | (ALS or "Amyotrophic lateral sclerosis").mp. or exp Motor Neuron Disease/ or Motor neuron* disease*.mp. or Motor neuron disease*.mp. or MND.mp. or Charcot* disease.mp. or "Charcot* disease".mp. or anterior horn cell disease*.mp. or Lateral sclerosis.mp. or motor system disease*.mp. or primary lateral sclerosis.mp. or PLS.mp. or progressive muscular atrophy.mp. or PMA.mp. or progressive bulbar palsy.mp. or PBP.mp. or "Lou Gehrig* disease".mp. | 168,364 |
| 4 | #1 AND #2 AND #3 | 2004 |

**PsycINFO via Ovid**

**1806 to 23rd November 2023**

24th November 2023

| **#** | **Search Terms** | **Results** |
| --- | --- | --- |
| 1 | (HR-PRO or HRPRO or HRQL or HRQoL or QL or QoL).ti,ab. or quality of life.mp. or (health index* or health indices or health profile*).ti,ab. or health status.mp. or ((patient or self or child or parent or carer or proxy) adj (appraisal* or appraised or report or reported or reporting or rated or rating* or based or assessed or assessment*)).ti,ab. or ((disability or function or functional or functions or subjective or utility or utilities or wellbeing or well being) adj2 (index or indices or instrument or instruments or measure or measures or questionnaire* or profile or profiles or scale or scales or score or scores or status or survey or surveys)).ti,ab. | 345,331 |
| 2 | exp Caregivers/ or carer*.mp. or caring.mp. or carergiv*.mp. or care-giv*.mp. or spillover*.mp. or spill over*.mp. or partner*.mp. or husband.mp. or wife.mp. or spouse*.mp. or spousal.mp. or child.mp. or children.mp. or son.mp. or sons.mp. or daughter*.mp. or offspring*.mp. or parent*.mp. or mother*.mp. or maternal.mp. or father*.mp. or paternal.mp. or sibling*.mp. or brother*.mp. or sister*.mp. or dependent*.mp. or "next of kin".mp. or kinship.mp. or grandparent*.mp. or grandmother*.mp. or grandfather*.mp. or relative*.mp. or family.mp. or "close person*".mp. or families.mp. | 1,749,224 |
| 3 | exp Amyotrophic Lateral Sclerosis/ or ALS.mp. or "Amyotrophic lateral sclerosis".mp. or Motor neuron* disease*.mp. or Motor neuron disease*.mp. or MND.mp. or Charcot* disease.mp. or "Charcot* disease".mp. or anterior horn cell disease*.mp. or Lateral sclerosis.mp. or motor system disease*.mp. or primary lateral sclerosis.mp. or PLS.mp. or progressive muscular atrophy.mp. or PMA.mp. or progressive bulbar palsy.mp. or PBP.mp. or "Lou Gehrig* disease".mp. | 27,685 |
| 4 | #1 AND #2 AND #3 | 632 |

**CINAHL via EBSCO**

**1974 to 23rd November 2023**

24th November 2023

| **#** | **Search Terms** | **Results** |
| --- | --- | --- |
| 1 | TI ( ("HR-PRO" or "HRPRO" or "HRQL" or "HRQoL" or "QL" or "QoL" or “quality of life” or “health index*” or “health indices” or “health profile*” or “health status”) ) OR AB ( ("HR-PRO" or "HRPRO" or "HRQL" or "HRQoL" or "QL" or "QoL" or “quality of life” or “health index*” or “health indices” or “health profile*” or “health status”) ) | 187,099 |
| 2 | TI ( ((patient or self or child or parent or carer or proxy) N1 (appraisal* or appraised or report or reported or reporting or rated or rating* or based or assessed or assessment*)) ) OR AB ( ((patient or self or child or parent or carer or proxy) N1 (appraisal* or appraised or report or reported or reporting or rated or rating* or based or assessed or assessment*)) ) | 252,288 |
| 3 | TI ( ((disability or function or functional or functions or subjective or utility or utilities or wellbeing or well being) N2 (index or indices or instrument or instruments or measure or measures or questionnaire* or profile or profiles or scale or scales or score or scores or status or survey or surveys)) ) OR AB ( ((disability or function or functional or functions or subjective or utility or utilities or wellbeing or well being) N2 (index or indices or instrument or instruments or measure or measures or questionnaire* or profile or profiles or scale or scales or score or scores or status or survey or surveys)) ) | 82,748 |
| 4 | #1 OR #2 OR #3 | 465,301 |
| 5 | (MH "Caregivers") OR carer* OR caring OR carergiv* OR care-giv* OR spillover* OR spill over* OR partner* OR husband OR wife OR spouse* OR spousal OR child OR children OR son OR sons OR daughter* OR offspring* OR parent* OR mother* OR maternal OR father* OR paternal OR sibling* OR brother* OR sister* OR dependent* OR “next of kin” OR kinship OR grandparent* OR grandmother* OR grandfather* OR relative* OR family OR “close person*” OR families | 1,744,287 |
| 6 | (MH "Motor Neuron Diseases+") OR ALS OR “Amyotrophic lateral sclerosis” OR Motor neuron* disease* OR Motor neuron disease* OR MND OR Charcot* disease OR “Charcot* disease” OR anterior horn cell disease* OR Lateral sclerosis OR motor system disease* OR primary lateral sclerosis OR PLS OR progressive muscular atrophy OR PMA OR progressive bulbar palsy OR PBP OR “Lou Gehrig* disease” | 21,929 |
| 7 | #4 AND #5 AND #6 | 656 |

**Cochrane Database of Systematic Reviews via Wiley**

**1996 to 23rd November 2023**

24th November 2023

| **#** | **Search Terms** | **Results** |
| --- | --- | --- |
| 1 | (HR-PRO or HRPRO or HRQL or HRQoL or QL or QoL or “quality of life” or “health index” or “health indices” or “health profile” or "health status"):ti,ab,kw | 162,519 |
| 2 | ((patient or self or child or parent or carer or proxy) NEXT/1 (appraisal* or appraised or report or reported or reporting or rated or rating* or based or assessed or assessment*)):ti,ab,kw | 79,452 |
| 3 | ((disability or function or functional or functions or subjective or utility or utilities or wellbeing or well being) NEXT/2 (index or indices or instrument or instruments or measure or measures or questionnaire* or profile or profiles or scale or scales or score or scores or status or survey or surveys)):ti,ab,kw | 50,135 |
| 4 | #1 OR #2 OR #3 | 252,114 |
| 5 | MeSH descriptor: [Caregivers] explode all trees | 58 |
| 6 | (carer* OR caring OR carergiv* OR care-giv* OR spillover* OR spill over* OR partner* OR husband OR wife OR spouse* OR spousal OR child OR children OR son OR sons OR daughter* OR offspring* OR parent* OR mother* OR maternal OR father* OR paternal OR sibling* OR brother* OR sister* OR dependent* OR “next of kin” OR kinship OR grandparent* OR grandmother* OR grandfather* OR relative* OR family OR “close person” OR families):ti,ab,kw | 7,571 |
| 7 | #5 OR #6 | 7,574 |
| 8 | MeSH descriptor: [Motor Neuron Disease] explode all trees | 27 |
| 9 | (ALS OR “Amyotrophic lateral sclerosis” OR “Motor neuron disease” OR “Motor neurone disease” OR MND OR “Charcot’s disease” OR “Charcot disease” OR “anterior horn cell disease” OR “Lateral sclerosis” OR “motor system disease” OR “primary lateral sclerosis” OR PLS OR “progressive muscular atrophy” OR PMA OR “progressive bulbar palsy” OR PBP OR “Lou Gehrig disease”):ti,ab,kw | 78 |
| 10 | #8 OR #9 | 81 |
| 11 | #4 AND #7 AND #10 | 23 |

**Cochrane Central Register of Controlled Trials via Wiley**

**1898 to 23rd November 2023**

24th November 2023

| **#** | **Search Terms** | **Results** |
| --- | --- | --- |
| 1 | (HR-PRO or HRPRO or HRQL or HRQoL or QL or QoL or “quality of life” or “health index” or “health indices” or “health profile” or "health status"):ti,ab,kw | 162,519 |
| 2 | ((patient or self or child or parent or carer or proxy) NEXT/1 (appraisal* or appraised or report or reported or reporting or rated or rating* or based or assessed or assessment*)):ti,ab,kw | 79,452 |
| 3 | ((disability or function or functional or functions or subjective or utility or utilities or wellbeing or well being) NEXT/2 (index or indices or instrument or instruments or measure or measures or questionnaire* or profile or profiles or scale or scales or score or scores or status or survey or surveys)):ti,ab,kw | 50,135 |
| 4 | #1 OR #2 OR #3 | 252,114 |
| 5 | MeSH descriptor: [Caregivers] explode all trees | 3,204 |
| 6 | (carer* OR caring OR carergiv* OR care-giv* OR spillover* OR spill over* OR partner* OR husband OR wife OR spouse* OR spousal OR child OR children OR son OR sons OR daughter* OR offspring* OR parent* OR mother* OR maternal OR father* OR paternal OR sibling* OR brother* OR sister* OR dependent* OR “next of kin” OR kinship OR grandparent* OR grandmother* OR grandfather* OR relative* OR family OR “close person” OR families):ti,ab,kw | 418,282 |
| 7 | #5 OR #6 | 418,958 |
| 8 | MeSH descriptor: [Motor Neuron Disease] explode all trees | 1,024 |
| 9 | (ALS OR “Amyotrophic lateral sclerosis” OR “Motor neuron disease” OR “Motor neurone disease” OR MND OR “Charcot’s disease” OR “Charcot disease” OR “anterior horn cell disease” OR “Lateral sclerosis” OR “motor system disease” OR “primary lateral sclerosis” OR PLS OR “progressive muscular atrophy” OR PMA OR “progressive bulbar palsy” OR PBP OR “Lou Gehrig disease”):ti,ab,kw | 3,818 |
| 10 | #8 OR #9 | 3940 |
| 11 | #4 AND #7 AND #10 | 236 |

**Stage 2 Search**

| **Host** | **Database** | **Dates Covered** | **Date Searched** | **Search Yield** |
| --- | --- | --- | --- | --- |
| Ovid | MEDLINE(R) and Epub Ahead of Print, In-Process & Other Non-Indexed Citations, Daily and Versions(R) | 1946 - Date | 04/04/2024 | 1,108 |
| Ovid | Embase | 1974 - Date | 04/04/2024 | 445 |
| Ovid | PsycINFO | 1806 - Date | 04/04/2024 | 835 |
| EBSCO | CINAHL | 1974 - Date | 04/04/2024 | 296 |
| Wiley | Cochrane Database of Systematic Reviews | 1996 - Date | 04/04/2024 | 0 |
| Wiley | Cochrane Central Register of Controlled Trials | 1898 - Date | 04/04/2024 | 102 |
| **TOTAL** | | | | **2786** |
| Google | Google Scholar (First 100 results screened) | | 22/04/2024 | 4379 |

No restrictions to the publication date were applied to articles. Databases were searched separately with specific search criteria:

Psychometric Properties Filter Source

<https://www.cosmin.nl/tools/PubMed-search-filters/> [Accessed on: 03/04/2024]

**Ovid MEDLINE(R) and Epub Ahead of Print, In-Process & Other Non-Indexed Citations, Daily and Versions(R)**

**1946 to 2nd April 2024**

4th April 2024

| **#** | **Search Terms** | **Results** |
| --- | --- | --- |
| 1 | exp Caregivers/ or carer*.mp. or caring.mp. or carergiv*.mp. or care-giv*.mp. or spillover*.mp. or spill over*.mp. or partner*.mp. or husband.mp. or wife.mp. or spouse*.mp. or spousal.mp. or child.mp. or children.mp. or son.mp. or sons.mp. or daughter*.mp. or offspring*.mp. or parent*.mp. or mother*.mp. or maternal.mp. or father*.mp. or paternal.mp. or sibling*.mp. or brother*.mp. or sister*.mp. or dependent*.mp. or "next of kin".mp. or kinship.mp. or grandparent*.mp. or grandmother*.mp. or grandfather*.mp. or relative*.mp. or family.mp. or "close person*".mp. or families.mp. | 7,719,193 |
| 2 | (ALS or "Amyotrophic lateral sclerosis").mp. or exp Motor Neuron Disease/ or Motor neuron* disease*.mp. or Motor neuron disease*.mp. or MND.mp. or Charcot* disease.mp. or "Charcot* disease".mp. or anterior horn cell disease*.mp. or Lateral sclerosis.mp. or motor system disease*.mp. or primary lateral sclerosis.mp. or PLS.mp. or progressive muscular atrophy.mp. or PMA.mp. or progressive bulbar palsy.mp. or PBP.mp. or "Lou Gehrig* disease".mp. | 143,733 |
| 3 | 1 AND 2 | 35,489 |
| 4 | (“Acceptance of illness” or ais).mp. | 19,523 |
| 5 | (“ALS Depression Inventory 12” or “ALS Depression Inventory” or ADI-12).mp. | 23 |
| 6 | (“beck depression inventory” or “beck inventory” or bdi).mp. | 18,788 |
| 7 | (“Beck Hopelessness Scale” or bhs).mp. | 1,606 |
| 8 | (“Brief symptom inventory” or bsi).mp. | 7,217 |
| 9 | (“burden scale for family caregivers” or bsfc).mp. | 173 |
| 10 | (“caregiver burden inventory” or cbi).mp. | 1,850 |
| 11 | (“caregiver burden scale” or cbs).mp. | 10,472 |
| 12 | (“caregiver network scale” or cns).mp. | 129,138 |
| 13 | (“caregiver strain index” or csi).mp. | 5,269 |
| 14 | (“carer quality of life” or Carerqol).mp. | 136 |
| 15 | (“Center for Epidemiology Articles Depression Scale” or CES-D-10).mp. | 263 |
| 16 | (“chalder fatigue scale”).mp. | 224 |
| 17 | (“close person questionnaire” or cpq).mp. | 387 |
| 18 | (“Coping Inventory for Stressful Situations” or CISS).mp. | 1,439 |
| 19 | (“Coping Orientation to Problems Experienced Inventory” or “brief cope”).mp. | 933 |
| 20 | (“cost of care index” or cci).mp. | 10,282 |
| 21 | (“depression anxiety and stress scale” or dass or dass-21).mp. | 10,721 |
| 22 | (“dyadic adjustment scale” or das).mp. | 75,492 |
| 23 | (“euroqol-5 dimensions” or eq5d5l).mp. | 1,214 |
| 24 | (“Folkham's Ways of Coping Scale” or wocq).mp. | 9 |
| 25 | (“Functional Assessment of Chronic Illness Therapy–Spiritual Well-Being Scale” or facit-sp).mp. | 267 |
| 26 | (“General Health Questionnaire” or ghq or ghq-12 or ghq12).mp. | 6,917 |
| 27 | (“Hamilton Anxiety Rating Scale” or hars).mp. | 1,732 |
| 28 | (“Hamilton Depression Rating Scale“ or hdrs).mp. | 6,634 |
| 29 | (“Hospital Anxiety and Depression Scale” or hads).mp. | 14,413 |
| 30 | (“Life Satisfaction Checklist” or LiSatt-11).mp. | 44 |
| 31 | (“McGill Quality of Life Questionnaire” or MQol).mp. | 203 |
| 32 | (“metacognitive questionnaire 30” or MCQ-30).mp. | 129 |
| 33 | (“Multidimensional Scale of Perceived Social Support” or mspss).mp. | 1,269 |
| 34 | (“Patient Health Questionnaire 9” or phq9 or phq-9).mp. | 10,197 |
| 35 | (“Positive and Negative Affect Schedule” or panas).mp. | 1,335 |
| 36 | (“profile of mood states” or poms or poms-sf or “pomssf”).mp. | 4,533 |
| 37 | (“purpose in life test” or pil).mp. | 1,691 |
| 38 | (“Quality of Life at the End of Life” or QUAL-E-fam).mp. | 120 |
| 39 | (“Quality of Life Enjoyment & Satisfaction Questionnaire Short Form” or Q-LES-Q-SF).mp. | 86 |
| 40 | (“Quality of Life in Life-Threatening Illness Family Carer Version 2” or QOLLTI-F).mp. | 15 |
| 41 | (“Rand 36-Item Health Survey” or rand-36).mp. | 1,181 |
| 42 | (“Satisfaction With Life Scale” or swls).mp. | 1,747 |
| 43 | (“Self-Rating Anxiety Scale” or SAS).mp. | 17,333 |
| 44 | (“Self-Rating Depression Scale” or sds).mp. | 86,289 |
| 45 | (“Short Form 12” or sf-12 or sf12).mp. | 8,056 |
| 46 | (“Short Form 36” or sf-36 or sf36).mp. | 31,761 |
| 47 | (“Short Form 8” or sf-8 or sf8).mp. | 778 |
| 48 | (“State-Trait Anxiety Inventory” or staix or staiy or stai).mp. | 8,359 |
| 49 | (“The Duke-UNC Functional Social Support Questionnaire” or fssq).mp. | 70 |
| 50 | (“Ways of Coping Checklist” or wocc).mp. | 161 |
| 51 | (“World Health Organization Quality of Life-BREF” or whoqol-bref or whoqolbref).mp. | 3,613 |
| 52 | (“Zarit Burden Interview” or zbi).mp. | 1,090 |
| 53 | (“Zarit Caregiver Burden Scale”).mp. | 96 |
| 54 | (“Zung Depression Scale” or zds).mp. | 405 |
| 55 | OR/4-54 | 480,133 |
| 56 | 3 AND 55 | 3588 |
| 57 | (instrumentation or methods).sh. | 231,755 |
| 58 | (validation study or comparative study).pt. | 2,002,880 |
| 59 | exp Psychometrics/ | 90,821 |
| 60 | psychometr*.tw. | 63,907 |
| 61 | (clinimetr* or clinometr*).mp. | 1,652 |
| 62 | exp Outcome Assessment, Health Care/ | 1,373,232 |
| 63 | outcome assessment.tw. | 5,208 |
| 64 | outcome measure*.mp. | 299,777 |
| 65 | exp Observer Variation/ | 45,189 |
| 66 | observer variation.tw. | 1,219 |
| 67 | exp Health Status Indicators/ | 345,398 |
| 68 | exp Reproducibility of Results/ | 478,127 |
| 69 | reproducib*.tw. | 197,343 |
| 70 | exp Discriminant Analysis/ | 12,061 |
| 71 | (reliab* or unreliab* or valid* or coefficient of variation or coefficient or homogeneity or homogeneous or internal consistency).tw. | 1,926,183 |
| 72 | (cronbach* and (alpha or alphas)).tw. | 33,867 |
| 73 | (item and (correlation* or selection* or reduction*)).tw. | 31,855 |
| 74 | (agreement or precision or imprecision or “precise values” or test-retest).mp. | 583,564 |
| 75 | (test and retest).tw. | 36,272 |
| 76 | (reliab* and (test or retest)).tw. | 119,655 |
| 77 | (stability or interrater or inter-rater or intrarater or intra-rater).tw. | 625,314 |
| 78 | (intertester or inter-tester or intratester or intra-tester).tw. | 686 |
| 79 | (interobserver or inter-observer or intraobserver or intra-observer).tw. | 35,571 |
| 80 | (intertechnician or inter-technician or intratechnician or intra-technician).tw. | 25 |
| 81 | (interexaminer or inter-examiner or intraexaminer or intra-examiner).tw. | 2,501 |
| 82 | (interassay or inter-assay or intraassay or intra-assay).tw. | 10,193 |
| 83 | (interindividual or inter-individual or intraindividual or intra-individual).tw. | 42,987 |
| 84 | (interparticipant or inter-participant or intraparticipant or intra-participant).tw. | 221 |
| 85 | (kappa or kappas).tw. | 118,050 |
| 86 | ((repeatab* or replicab* or repeated) and (measure or measures or findings or result or results or test or tests)).mp. | 283,551 |
| 87 | (generaliza* or generalisa*).tw. | 67,538 |
| 88 | concordance.tw. | 61,874 |
| 89 | (intraclass and correlation*).tw. | 36,036 |
| 90 | discriminative.tw. | 26,561 |
| 91 | known group.tw. | 1,644 |
| 92 | (factor analysis or factor analyses or factor structure or factor structures).tw. | 72,305 |
| 93 | (dimension* or subscale).tw. | 773,449 |
| 94 | (multitrait and scaling and (analysis or analyses)).tw. | 157 |
| 95 | item discriminant.tw. | 131 |
| 96 | interscale correlation*.tw. | 169 |
| 97 | (error or errors).tw. | 395,631 |
| 98 | individual variability.tw. | 11,202 |
| 99 | interval variability.tw. | 714 |
| 100 | rate variability.tw. | 24,327 |
| 101 | (variability and (analysis or values)).tw. | 129,060 |
| 102 | (uncertainty and (measurement or measuring)).tw. | 10,758 |
| 103 | standard error of measurement.tw. | 2,814 |
| 104 | sensitiv*.tw. | 1,715,047 |
| 105 | responsive*.tw. | 284,343 |
| 106 | (limit and detection).tw. | 124,887 |
| 107 | minimal detectable concentration.tw. | 86 |
| 108 | interpretab*.tw. | 18,787 |
| 109 | ((minimal or minimally or clinical or clinically) and (important or significant or detectable) and (change or difference)).tw. | 324,749 |
| 110 | (small* and (real or detectable) and (change or difference)).tw. | 9,870 |
| 111 | meaningful change.tw. | 1,651 |
| 112 | ceiling effect.tw. | 2,438 |
| 113 | floor effect.tw. | 861 |
| 114 | item response model.tw. | 176 |
| 115 | IRT.tw. | 4,631 |
| 116 | rasch.tw. | 5,699 |
| 117 | differential item functioning.tw. | 2,453 |
| 118 | DIF.tw. | 3,551 |
| 119 | computer adaptive testing.tw. | 265 |
| 120 | item bank.tw. | 838 |
| 121 | cross-cultural equivalence.tw. | 149 |
| 122 | OR/57-121 | 9,215,837 |
| 123 | 56 AND 122 | 1,108 |

**Embase via Ovid**

**1946 to 3rd April 2024**

4th April 2024

| **#** | **Search Terms** | **Results** |
| --- | --- | --- |
| 1 | (carer* or caring or carergiv* or care-giv* or spillover* or spill over* or partner* or husband or wife or spouse* or spousal or child or children or son or sons or daughter* or offspring* or parent* or mother* or maternal or father* or paternal or sibling* or brother* or sister* or dependent* or "next of kin" or kinship or grandparent* or grandmother* or grandfather* or relative* or family or "close person*" or families).mp. | 9,586,307 |
| 2 | (ALS or "Amyotrophic lateral sclerosis").mp. or exp Motor Neuron Disease/ or Motor neuron* disease*.mp. or Motor neuron disease*.mp. or MND.mp. or Charcot* disease.mp. or "Charcot* disease".mp. or anterior horn cell disease*.mp. or Lateral sclerosis.mp. or motor system disease*.mp. or primary lateral sclerosis.mp. or PLS.mp. or progressive muscular atrophy.mp. or PMA.mp. or progressive bulbar palsy.mp. or PBP.mp. or "Lou Gehrig* disease".mp. | 171,577 |
| 3 | 1 AND 2 | 43,094 |
| 4 | (“Acceptance of illness” or ais).mp. | 30,726 |
| 5 | (“ALS Depression Inventory 12” or “ALS Depression Inventory” or ADI-12).mp. | 39 |
| 6 | (“beck depression inventory” or “beck inventory” or bdi).mp. | 42,237 |
| 7 | (“Beck Hopelessness Scale” or bhs).mp. | 2,678 |
| 8 | (“Brief symptom inventory” or bsi).mp. | 12,226 |
| 9 | (“burden scale for family caregivers” or bsfc).mp. | 180 |
| 10 | (“caregiver burden inventory” or cbi).mp. | 2,620 |
| 11 | (“caregiver burden scale” or cbs).mp. | 13,246 |
| 12 | (“caregiver network scale” or cns).mp. | 193,516 |
| 13 | (“caregiver strain index” or csi).mp. | 8,183 |
| 14 | (“carer quality of life” or Carerqol).mp. | 207 |
| 15 | (“Center for Epidemiology Articles Depression Scale” or CES-D-10).mp. | 359 |
| 16 | (“chalder fatigue scale”).mp. | 487 |
| 17 | (“close person questionnaire” or cpq).mp. | 453 |
| 18 | (“Coping Inventory for Stressful Situations” or CISS).mp. | 1,916 |
| 19 | (“Coping Orientation to Problems Experienced Inventory” or “brief cope”).mp. | 1,533 |
| 20 | (“cost of care index” or cci).mp. | 20,509 |
| 21 | (“depression anxiety and stress scale” or dass or dass-21).mp. | 8,546 |
| 22 | (“dyadic adjustment scale” or das).mp. | 64,667 |
| 23 | (“euroqol-5 dimensions” or eq5d5l).mp. | 1,746 |
| 24 | (“Folkham's Ways of Coping Scale” or wocq).mp. | 17 |
| 25 | (“Functional Assessment of Chronic Illness Therapy–Spiritual Well-Being Scale” or facit-sp).mp. | 454 |
| 26 | (“General Health Questionnaire” or ghq or ghq-12 or ghq12).mp. | 10,029 |
| 27 | (“Hamilton Anxiety Rating Scale” or hars).mp. | 2,814 |
| 28 | (“Hamilton Depression Rating Scale“ or hdrs).mp. | 20,580 |
| 29 | (“Hospital Anxiety and Depression Scale” or hads).mp. | 32,748 |
| 30 | (“Life Satisfaction Checklist” or LiSatt-11).mp. | 66 |
| 31 | (“McGill Quality of Life Questionnaire” or MQol).mp. | 338 |
| 32 | (“metacognitive questionnaire 30” or MCQ-30).mp. | 169 |
| 33 | (“Multidimensional Scale of Perceived Social Support” or mspss).mp. | 2,065 |
| 34 | (“Patient Health Questionnaire 9” or phq9 or phq-9).mp. | 21,571 |
| 35 | (“Positive and Negative Affect Schedule” or panas).mp. | 3,475 |
| 36 | (“profile of mood states” or poms or poms-sf or “pomssf”).mp. | 6,444 |
| 37 | (“purpose in life test” or pil).mp. | 2,236 |
| 38 | (“Quality of Life at the End of Life” or QUAL-E-fam).mp. | 171 |
| 39 | (“Quality of Life Enjoyment & Satisfaction Questionnaire Short Form” or Q-LES-Q-SF).mp. | 152 |
| 40 | (“Quality of Life in Life-Threatening Illness Family Carer Version 2” or QOLLTI-F).mp. | 19 |
| 41 | (“Rand 36-Item Health Survey” or rand-36).mp. | 1,643 |
| 42 | (“Satisfaction With Life Scale” or swls).mp. | 2,707 |
| 43 | (“Self-Rating Anxiety Scale” or SAS).mp. | 101,311 |
| 44 | (“Self-Rating Depression Scale” or sds).mp. | 110,358 |
| 45 | (“Short Form 12” or sf-12 or sf12).mp. | 15,767 |
| 46 | (“Short Form 36” or sf-36 or sf36).mp. | 62,496 |
| 47 | (“Short Form 8” or sf-8 or sf8).mp. | 1,448 |
| 48 | (“State-Trait Anxiety Inventory” or staix or staiy or stai).mp. | 16,521 |
| 49 | (“The Duke-UNC Functional Social Support Questionnaire” or fssq).mp. | 108 |
| 50 | (“Ways of Coping Checklist” or wocc).mp. | 229 |
| 51 | (“World Health Organization Quality of Life-BREF” or whoqol-bref or whoqolbref).mp. | 5,840 |
| 52 | (“Zarit Burden Interview” or zbi).mp. | 1,779 |
| 53 | (“Zarit Caregiver Burden Scale”).mp. | 150 |
| 54 | (“Zung Depression Scale” or zds).mp. | 638 |
| 55 | OR/4-54 | 779,960 |
| 56 | 3 AND 55 | 1,844 |
| 57 | (instrumentation or methods).sh. | 180,638 |
| 58 | (“validation study” or “comparative study”).tw. | 137,825 |
| 59 | exp Psychometrics/ | 115,181 |
| 60 | psychometr*.tw. | 76,072 |
| 61 | (clinimetr* or clinometr*).mp. | 2,447 |
| 62 | exp “Outcome Assessment, Health Care”/ | 910,308 |
| 63 | “outcome assessment”.tw. | 7,219 |
| 64 | “outcome measure*”.mp. | 376,695 |
| 65 | exp “Observer Variation”/ | 21,190 |
| 66 | “observer variation”.tw. | 1,835 |
| 67 | exp “Health Status Indicators”/ | 45,374 |
| 68 | exp “Reproducibility of Results”/ | 266,959 |
| 69 | reproducib*.tw. | 252,639 |
| 70 | exp “Discriminant Analysis”/ | 28,772 |
| 71 | (reliab* or unreliab* or valid* or coefficient of variation or coefficient or homogeneity or homogeneous or internal consistency).tw. | 2,519,048 |
| 72 | (cronbach* and (alpha or alphas)).tw. | 39,994 |
| 73 | (item and (correlation* or selection* or reduction*)).tw. | 43,292 |
| 74 | (agreement or precision or imprecision or “precise values” or test-retest).mp. | 705,310 |
| 75 | (test and retest).tw. | 43,607 |
| 76 | (reliab* and (test or retest)).tw. | 158,021 |
| 77 | (stability or interrater or inter-rater or intrarater or intra-rater).tw. | 700,891 |
| 78 | (intertester or inter-tester or intratester or intra-tester).tw. | 843 |
| 79 | (interobserver or inter-observer or intraobserver or intra-observer).tw. | 49,223 |
| 80 | (intertechnician or inter-technician or intratechnician or intra-technician).tw. | 45 |
| 81 | (interexaminer or inter-examiner or intraexaminer or intra-examiner).tw. | 2,589 |
| 82 | (interassay or inter-assay or intraassay or intra-assay).tw. | 13,946 |
| 83 | (interindividual or inter-individual or intraindividual or intra-individual).tw. | 55,573 |
| 84 | (interparticipant or inter-participant or intraparticipant or intra-participant).tw. | 274 |
| 85 | (kappa or kappas).tw. | 141,645 |
| 86 | ((repeatab* or replicab* or repeated) and (measure or measures or findings or result or results or test or tests)).mp. | 413,295 |
| 87 | (generaliza* or generalisa*).tw. | 77,713 |
| 88 | concordance.tw. | 95,851 |
| 89 | (intraclass and correlation*).tw. | 43,284 |
| 90 | discriminative.tw. | 33,507 |
| 91 | known group.tw. | 1,920 |
| 92 | (factor analysis or factor analyses or factor structure or factor structures).tw. | 82,227 |
| 93 | (dimension* or subscale).tw. | 831,995 |
| 94 | (multitrait and scaling and (analysis or analyses)).tw. | 164 |
| 95 | item discriminant.tw. | 142 |
| 96 | interscale correlation*.tw. | 182 |
| 97 | (error or errors).tw. | 517,811 |
| 98 | individual variability.tw. | 15,488 |
| 99 | interval variability.tw. | 914 |
| 100 | rate variability.tw. | 34,012 |
| 101 | (variability and (analysis or values)).tw. | 180,298 |
| 102 | (uncertainty and (measurement or measuring)).tw. | 12,204 |
| 103 | standard error of measurement.tw. | 3,284 |
| 104 | sensitiv*.tw. | 2,156,585 |
| 105 | responsive*.tw. | 344,256 |
| 106 | (limit and detection).tw. | 142,998 |
| 107 | minimal detectable concentration.tw. | 109 |
| 108 | interpretab*.tw. | 22,576 |
| 109 | ((minimal or minimally or clinical or clinically) and (important or significant or detectable) and (change or difference)).tw. | 560,453 |
| 110 | (small* and (real or detectable) and (change or difference)).tw. | 16,116 |
| 111 | meaningful change.tw. | 2,868 |
| 112 | ceiling effect.tw. | 3,361 |
| 113 | floor effect.tw. | 1,182 |
| 114 | item response model.tw. | 167 |
| 115 | IRT.tw. | 5,915 |
| 116 | rasch.tw. | 6,756 |
| 117 | differential item functioning.tw. | 2,723 |
| 118 | DIF.tw. | 6,184 |
| 119 | computer adaptive testing.tw. | 382 |
| 120 | item bank.tw. | 1,148 |
| 121 | cross-cultural equivalence.tw. | 166 |
| 122 | OR/57-121 | 8,953,504 |
| 123 | 56 AND 122 | 445 |

**PsycINFO via Ovid**

**1806 to 3rd April 2024**

4th April 2024

| **#** | **Search Terms** | **Results** |
| --- | --- | --- |
| 1 | exp Caregivers/ or carer*.mp. or caring.mp. or carergiv*.mp. or care-giv*.mp. or spillover*.mp. or spill over*.mp. or partner*.mp. or husband.mp. or wife.mp. or spouse*.mp. or spousal.mp. or child.mp. or children.mp. or son.mp. or sons.mp. or daughter*.mp. or offspring*.mp. or parent*.mp. or mother*.mp. or maternal.mp. or father*.mp. or paternal.mp. or sibling*.mp. or brother*.mp. or sister*.mp. or dependent*.mp. or "next of kin".mp. or kinship.mp. or grandparent*.mp. or grandmother*.mp. or grandfather*.mp. or relative*.mp. or family.mp. or "close person*".mp. or families.mp. | 1,771,516 |
| 2 | exp Amyotrophic Lateral Sclerosis/ or ALS.mp. or "Amyotrophic lateral sclerosis".mp. or Motor neuron* disease*.mp. or Motor neuron disease*.mp. or MND.mp. or Charcot* disease.mp. or "Charcot* disease".mp. or anterior horn cell disease*.mp. or Lateral sclerosis.mp. or motor system disease*.mp. or primary lateral sclerosis.mp. or PLS.mp. or progressive muscular atrophy.mp. or PMA.mp. or progressive bulbar palsy.mp. or PBP.mp. or "Lou Gehrig* disease".mp. | 28,579 |
| 3 | 1 AND 2 | 8,263 |
| 4 | (“Acceptance of illness” or ais).mp. | 1,951 |
| 5 | (“ALS Depression Inventory 12” or “ALS Depression Inventory” or ADI-12).mp. | 12 |
| 6 | (“beck depression inventory” or “beck inventory” or bdi).mp. | 52,146 |
| 7 | (“Beck Hopelessness Scale” or bhs).mp. | 2,677 |
| 8 | (“Brief symptom inventory” or bsi).mp. | 9,129 |
| 9 | (“burden scale for family caregivers” or bsfc).mp. | 59 |
| 10 | (“caregiver burden inventory” or cbi).mp. | 1,106 |
| 11 | (“caregiver burden scale” or cbs).mp. | 1,504 |
| 12 | (“caregiver network scale” or cns).mp. | 30,150 |
| 13 | (“caregiver strain index” or csi).mp. | 1,321 |
| 14 | (“carer quality of life” or Carerqol).mp. | 86 |
| 15 | (“Center for Epidemiology Articles Depression Scale” or CES-D-10).mp. | 219 |
| 16 | (“chalder fatigue scale”).mp. | 241 |
| 17 | (“close person questionnaire” or cpq).mp. | 222 |
| 18 | (“Coping Inventory for Stressful Situations” or CISS).mp. | 830 |
| 19 | (“Coping Orientation to Problems Experienced Inventory” or “brief cope”).mp. | 2,281 |
| 20 | (“cost of care index” or cci).mp. | 1,594 |
| 21 | (“depression anxiety and stress scale” or dass or dass-21).mp. | 8,509 |
| 22 | (“dyadic adjustment scale” or das).mp. | 20,002 |
| 23 | (“euroqol-5 dimensions” or eq5d5l).mp. | 299 |
| 24 | (“Folkham's Ways of Coping Scale” or wocq).mp. | 27 |
| 25 | (“Functional Assessment of Chronic Illness Therapy–Spiritual Well-Being Scale” or facit-sp).mp. | 167 |
| 26 | (“General Health Questionnaire” or ghq or ghq-12 or ghq12).mp. | 10,509 |
| 27 | (“Hamilton Anxiety Rating Scale” or hars).mp. | 5,077 |
| 28 | (“Hamilton Depression Rating Scale“ or hdrs).mp. | 7,257 |
| 29 | (“Hospital Anxiety and Depression Scale” or hads).mp. | 15,007 |
| 30 | (“Life Satisfaction Checklist” or LiSatt-11).mp. | 41 |
| 31 | (“McGill Quality of Life Questionnaire” or MQol).mp. | 254 |
| 32 | (“metacognitive questionnaire 30” or MCQ-30).mp. | 163 |
| 33 | (“Multidimensional Scale of Perceived Social Support” or mspss).mp. | 3,850 |
| 34 | (“Patient Health Questionnaire 9” or phq9 or phq-9).mp. | 13,376 |
| 35 | (“Positive and Negative Affect Schedule” or panas).mp. | 10,248 |
| 36 | (“profile of mood states” or poms or poms-sf or “pomssf”).mp. | 5,909 |
| 37 | (“purpose in life test” or pil).mp. | 776 |
| 38 | (“Quality of Life at the End of Life” or QUAL-E-fam).mp. | 94 |
| 39 | (“Quality of Life Enjoyment & Satisfaction Questionnaire Short Form” or Q-LES-Q-SF).mp. | 64 |
| 40 | (“Quality of Life in Life-Threatening Illness Family Carer Version 2” or QOLLTI-F).mp. | 7 |
| 41 | (“Rand 36-Item Health Survey” or rand-36).mp. | 854 |
| 42 | (“Satisfaction With Life Scale” or swls).mp. | 9,447 |
| 43 | (“Self-Rating Anxiety Scale” or SAS).mp. | 4,639 |
| 44 | (“Self-Rating Depression Scale” or sds).mp. | 6,747 |
| 45 | (“Short Form 12” or sf-12 or sf12).mp. | 3,127 |
| 46 | (“Short Form 36” or sf-36 or sf36).mp. | 11,088 |
| 47 | (“Short Form 8” or sf-8 or sf8).mp. | 436 |
| 48 | (“State-Trait Anxiety Inventory” or staix or staiy or stai).mp. | 24,048 |
| 49 | (“The Duke-UNC Functional Social Support Questionnaire” or fssq).mp. | 47 |
| 50 | (“Ways of Coping Checklist” or wocc).mp. | 642 |
| 51 | (“World Health Organization Quality of Life-BREF” or whoqol-bref or whoqolbref).mp. | 3,131 |
| 52 | (“Zarit Burden Interview” or zbi).mp. | 1,251 |
| 53 | (“Zarit Caregiver Burden Scale”).mp. | 80 |
| 54 | (“Zung Depression Scale” or zds).mp. | 216 |
| 55 | OR/4-54 | 230,300 |
| 56 | 3 AND 55 | 2,086 |
| 57 | (instrumentation or methods).tw. | 720,336 |
| 58 | (“validation study” or “comparative study”).tw. | 18,632 |
| 59 | exp Psychometrics/ | 236,841 |
| 60 | psychometr*.tw. | 93,530 |
| 61 | (clinimetr* or clinometr*).mp. | 392 |
| 62 | “outcome assessment”.tw. | 1,700 |
| 63 | “outcome measure*”.mp. | 47,112 |
| 64 | “observer variation”.tw. | 28 |
| 65 | reproducib*.tw. | 7,678 |
| 66 | (reliab* or unreliab* or valid* or coefficient of variation or coefficient or homogeneity or homogeneous or internal consistency).tw. | 433,908 |
| 67 | (cronbach* and (alpha or alphas)).tw. | 16,865 |
| 68 | (item and (correlation* or selection* or reduction*)).tw. | 23,363 |
| 69 | (agreement or precision or imprecision or “precise values” or test-retest).mp. | 87,850 |
| 70 | (test and retest).tw. | 23,119 |
| 71 | (reliab* and (test or retest)).tw. | 69,186 |
| 72 | (stability or interrater or inter-rater or intrarater or intra-rater).tw. | 64,903 |
| 73 | (intertester or inter-tester or intratester or intra-tester).tw. | 46 |
| 74 | (interobserver or inter-observer or intraobserver or intra-observer).tw. | 1,336 |
| 75 | (intertechnician or inter-technician or intratechnician or intra-technician).tw. | 0 |
| 76 | (interexaminer or inter-examiner or intraexaminer or intra-examiner).tw. | 103 |
| 77 | (interassay or inter-assay or intraassay or intra-assay).tw. | 42 |
| 78 | (interindividual or inter-individual or intraindividual or intra-individual).tw. | 11,823 |
| 79 | (interparticipant or inter-participant or intraparticipant or intra-participant).tw. | 96 |
| 80 | (kappa or kappas).tw. | 9,204 |
| 81 | ((repeatab* or replicab* or repeated) and (measure or measures or findings or result or results or test or tests)).mp. | 67,392 |
| 82 | (generaliza* or generalisa*).tw. | 48,771 |
| 83 | concordance.tw. | 7,854 |
| 84 | (intraclass and correlation*).tw. | 5,819 |
| 85 | discriminative.tw. | 11,089 |
| 86 | known group.tw. | 629 |
| 87 | (factor analysis or factor analyses or factor structure or factor structures).tw. | 86,059 |
| 88 | (dimension* or subscale).tw. | 227,249 |
| 89 | (multitrait and scaling and (analysis or analyses)).tw. | 71 |
| 90 | item discriminant.tw. | 56 |
| 91 | interscale correlation*.tw. | 145 |
| 92 | (error or errors).tw. | 135,108 |
| 93 | individual variability.tw. | 3,443 |
| 94 | interval variability.tw. | 109 |
| 95 | rate variability.tw. | 5,911 |
| 96 | (variability and (analysis or values)).tw. | 18,087 |
| 97 | (uncertainty and (measurement or measuring)).tw. | 1,518 |
| 98 | standard error of measurement.tw. | 740 |
| 99 | sensitiv*.tw. | 192,480 |
| 100 | responsive*.tw. | 50,958 |
| 101 | (limit and detection).tw. | 822 |
| 102 | minimal detectable concentration.tw. | 0 |
| 103 | interpretab*.tw. | 4,993 |
| 104 | ((minimal or minimally or clinical or clinically) and (important or significant or detectable) and (change or difference)).tw. | 35,385 |
| 105 | (small* and (real or detectable) and (change or difference)).tw. | 1,332 |
| 106 | meaningful change.tw. | 908 |
| 107 | ceiling effect.tw. | 1,112 |
| 108 | floor effect.tw. | 360 |
| 109 | item response model.tw. | 412 |
| 110 | IRT.tw. | 4,542 |
| 111 | rasch.tw. | 5,126 |
| 112 | differential item functioning.tw. | 3,084 |
| 113 | DIF.tw. | 2,318 |
| 114 | computer adaptive testing.tw. | 237 |
| 115 | item bank.tw. | 624 |
| 116 | cross-cultural equivalence.tw. | 203 |
| 117 | OR/57-116 | 1,776,390 |
| 118 | 56 AND 117 | 835 |

**CINAHL via EBSCO**

**1974 to 3th April 2024**

4th April 2024

| **#** | **Search Terms** | **Results** |
| --- | --- | --- |
| 1 | (MH "Caregivers") OR carer* OR caring OR carergiv* OR care-giv* OR spillover* OR spill over* OR partner* OR husband OR wife OR spouse* OR spousal OR child OR children OR son OR sons OR daughter* OR offspring* OR parent* OR mother* OR maternal OR father* OR paternal OR sibling* OR brother* OR sister* OR dependent* OR “next of kin” OR kinship OR grandparent* OR grandmother* OR grandfather* OR relative* OR family OR “close person*” OR families | 1,732,327 |
| 2 | (MH "Motor Neuron Diseases+") OR ALS OR “Amyotrophic lateral sclerosis” OR Motor neuron* disease* OR Motor neuron disease* OR MND OR Charcot* disease OR “Charcot* disease” OR anterior horn cell disease* OR Lateral sclerosis OR motor system disease* OR primary lateral sclerosis OR PLS OR progressive muscular atrophy OR PMA OR progressive bulbar palsy OR PBP OR “Lou Gehrig* disease” | 21,913 |
| 3 | 1 AND 2 | 5,854 |
| 4 | TI ( (“Acceptance of illness” or ais) ) OR AB ( (“Acceptance of illness” or ais) ) | 12,423 |
| 5 | TI ( (“ALS Depression Inventory 12” or “ALS Depression Inventory” or ADI-12) ) OR AB ( (“ALS Depression Inventory 12” or “ALS Depression Inventory” or ADI-12) ) | 3 |
| 6 | TI ( (“beck depression inventory” or “beck inventory” or bdi) ) OR AB ( (“beck depression inventory” or “beck inventory” or bdi) ) | 6,507 |
| 7 | TI ( (“Beck Hopelessness Scale” or bhs) ) OR AB ( (“Beck Hopelessness Scale” or bhs) ) | 556 |
| 8 | TI ( (“Brief symptom inventory” or bsi) ) OR AB ( (“Brief symptom inventory” or bsi) ) | 2,345 |
| 9 | TI ( (“burden scale for family caregivers” or bsfc) ) OR AB ( (“burden scale for family caregivers” or bsfc) ) | 38 |
| 10 | TI ( (“caregiver burden inventory” or cbi) ) OR AB ( (“caregiver burden inventory” or cbi) ) | 647 |
| 11 | TI ( (“caregiver burden scale” or cbs) ) OR AB ( (“caregiver burden scale” or cbs) ) | 1,068 |
| 12 | TI ( (“caregiver network scale” or cns) ) OR AB ( (“caregiver network scale” or cns) ) | 12,375 |
| 13 | TI ( (“caregiver strain index” or csi) ) OR AB ( (“caregiver strain index” or csi) ) | 1,232 |
| 14 | TI ( (“carer quality of life” or Carerqol) ) OR AB ( (“carer quality of life” or Carerqol) ) | 78 |
| 15 | TI ( (“Center for Epidemiology Articles Depression Scale” or CES-D-10) ) OR AB ( (“Center for Epidemiology Articles Depression Scale” or CES-D-10) ) | 87 |
| 16 | TI ( (“chalder fatigue scale”) ) OR AB ( (“chalder fatigue scale”) ) | 97 |
| 17 | TI ( (“close person questionnaire” or cpq) ) OR AB ( (“close person questionnaire” or cpq) ) | 138 |
| 18 | TI ( (“Coping Inventory for Stressful Situations” or CISS) ) OR AB ( (“Coping Inventory for Stressful Situations” or CISS) ) | 295 |
| 19 | TI ( (“Coping Orientation to Problems Experienced Inventory” or “brief cope”) ) OR AB ( (“Coping Orientation to Problems Experienced Inventory” or “brief cope”) ) | 433 |
| 20 | TI ( (“cost of care index” or cci) ) OR AB ( (“cost of care index” or cci) ) | 2,413 |
| 21 | TI ( (“depression anxiety and stress scale” or dass or dass-21) ) OR AB ( (“depression anxiety and stress scale” or dass or dass-21) ) | 3,515 |
| 22 | TI ( (“dyadic adjustment scale” or das) ) OR AB ( (“dyadic adjustment scale” or das) ) | 22,818 |
| 23 | TI ( (“euroqol-5 dimensions” or eq5d5l) ) OR AB ( (“euroqol-5 dimensions” or eq5d5l) ) | 175 |
| 24 | TI ( (“Folkham's Ways of Coping Scale” or wocq) ) OR AB ( (“Folkham's Ways of Coping Scale” or wocq) ) | 14 |
| 25 | TI ( (“Functional Assessment of Chronic Illness Therapy–Spiritual Well-Being Scale” or facit-sp) ) OR AB ( (“Functional Assessment of Chronic Illness Therapy–Spiritual Well-Being Scale” or facit-sp) ) | 172 |
| 26 | TI ( (“General Health Questionnaire” or ghq or ghq-12 or ghq12) ) OR AB ( (“General Health Questionnaire” or ghq or ghq-12 or ghq12) ) | 2,320 |
| 27 | TI ( (“Hamilton Anxiety Rating Scale” or hars) ) OR AB ( (“Hamilton Anxiety Rating Scale” or hars) ) | 1,234 |
| 28 | TI ( (“Hamilton Depression Rating Scale“ or hdrs) ) OR AB ( (“Hamilton Depression Rating Scale“ or hdrs) ) | 2,162 |
| 29 | TI ( (“Hospital Anxiety and Depression Scale” or hads) ) OR AB ( (“Hospital Anxiety and Depression Scale” or hads) ) | 891,439 |
| 30 | TI ( (“Life Satisfaction Checklist” or LiSatt-11) ) OR AB ( (“Life Satisfaction Checklist” or LiSatt-11) ) | 19 |
| 31 | TI ( (“McGill Quality of Life Questionnaire” or MQol) ) OR AB ( (“McGill Quality of Life Questionnaire” or MQol) ) | 128 |
| 32 | TI ( (“metacognitive questionnaire 30” or MCQ-30) ) OR AB ( (“metacognitive questionnaire 30” or MCQ-30) ) | 57 |
| 33 | TI ( (“Multidimensional Scale of Perceived Social Support” or mspss) ) OR AB ( (“Multidimensional Scale of Perceived Social Support” or mspss) ) | 728 |
| 34 | TI ( (“Patient Health Questionnaire 9” or phq9 or phq-9) ) OR AB ( (“Patient Health Questionnaire 9” or phq9 or phq-9) ) | 3,011 |
| 35 | TI ( (“Positive and Negative Affect Schedule” or panas) ) OR AB ( (“Positive and Negative Affect Schedule” or panas) ) | 566 |
| 36 | TI ( (“profile of mood states” or poms or poms-sf or “pomssf”) ) OR AB ( (“profile of mood states” or poms or poms-sf or “pomssf”) ) | 1,523 |
| 37 | TI ( (“purpose in life test” or pil) ) OR AB ( (“purpose in life test” or pil) ) | 272 |
| 38 | TI ( (“Quality of Life at the End of Life” or QUAL-E-fam) ) OR AB ( (“Quality of Life at the End of Life” or QUAL-E-fam) ) | 114 |
| 39 | TI ( (“Quality of Life Enjoyment & Satisfaction Questionnaire Short Form” or Q-LES-Q-SF) ) OR AB ( (“Quality of Life Enjoyment & Satisfaction Questionnaire Short Form” or Q-LES-Q-SF) ) | 34 |
| 40 | TI ( (“Quality of Life in Life-Threatening Illness Family Carer Version 2” or QOLLTI-F) ) OR AB ( (“Quality of Life in Life-Threatening Illness Family Carer Version 2” or QOLLTI-F) ) | 12 |
| 41 | TI ( (“Rand 36-Item Health Survey” or rand-36) ) OR AB ( (“Rand 36-Item Health Survey” or rand-36) ) | 445 |
| 42 | TI ( (“Satisfaction With Life Scale” or swls) ) OR AB ( (“Satisfaction With Life Scale” or swls) ) | 1,102 |
| 43 | TI ( (“Self-Rating Anxiety Scale” or SAS) ) OR AB ( (“Self-Rating Anxiety Scale” or SAS) ) | 3,784 |
| 44 | TI ( (“Self-Rating Depression Scale” or sds) ) OR AB ( (“Self-Rating Depression Scale” or sds) ) | 5,060 |
| 45 | TI ( (“Short Form 12” or sf-12 or sf12) ) OR AB ( (“Short Form 12” or sf-12 or sf12) ) | 3,265 |
| 46 | TI ( (“Short Form 36” or sf-36 or sf36) ) OR AB ( (“Short Form 36” or sf-36 or sf36) ) | 11,700 |
| 47 | TI ( (“Short Form 8” or sf-8 or sf8) ) OR AB ( (“Short Form 8” or sf-8 or sf8) ) | 246 |
| 48 | TI ( (“State-Trait Anxiety Inventory” or staix or staiy or stai) ) OR AB ( (“State-Trait Anxiety Inventory” or staix or staiy or stai) ) | 3,329 |
| 49 | TI ( (“The Duke-UNC Functional Social Support Questionnaire” or fssq) ) OR AB ( (“The Duke-UNC Functional Social Support Questionnaire” or fssq) ) | 33 |
| 50 | TI ( (“Ways of Coping Checklist” or wocc) ) OR AB ( (“Ways of Coping Checklist” or wocc) ) | 108 |
| 51 | TI ( (“World Health Organization Quality of Life-BREF” or whoqol-bref or whoqolbref) ) OR AB ( (“World Health Organization Quality of Life-BREF” or whoqol-bref or whoqolbref) ) | 1,785 |
| 52 | TI ( (“Zarit Burden Interview” or zbi) ) OR AB ( (“Zarit Burden Interview” or zbi) ) | 638 |
| 53 | TI ( (“Zarit Caregiver Burden Scale”) ) OR AB ( (“Zarit Caregiver Burden Scale”) ) | 61 |
| 54 | TI ( (“Zung Depression Scale” or zds) ) OR AB ( (“Zung Depression Scale” or zds) ) | 82 |
| 55 | S4 OR S5 OR S6 OR S7 OR S8 OR S9 OR S10 OR S11 OR S12 OR S13 OR S14 OR S15 OR S16 OR S17 OR S18 OR S19 OR S20 OR S21 OR S22 OR S23 OR S24 OR S25 OR S26 OR S27 OR S28 OR S29 OR S30 OR S31 OR S32 OR S33 OR S34 OR S35 OR S36 OR S37 OR S38 OR S39 OR S40 OR S41 OR S42 OR S43 OR S44 OR S45 OR S46 OR S47 OR S48 OR S49 OR S50 OR S51 OR S52 OR S53 OR S54 | 966,168 |
| 56 | 3 AND 55 | 1,914 |
| 57 | (MH “Psychometrics”) or ( TI psychometr* or AB psychometr* ) or ( TI clinimetr* or AB  clinimetr* ) or ( TI clinometr* OR AB clinometr* ) or (MH “Outcome Assessment”) or ( TI  outcome assessment or AB outcome assessment ) or ( TI outcome measure* or AB outcome measure* ) or (MH “Health Status Indicators”) or (MH “Reproducibility of Results”) or (MH “Discriminant Analysis”) or ( ( TI reproducib* or AB reproducib* ) or ( TI reliab* or AB reliab* ) or ( TI unreliab* or AB unreliab* ) ) or ( ( TI valid* or AB valid* ) or ( TI coefficient or AB coefficient ) or ( TI homogeneity or AB homogeneity ) ) or ( TI homogeneous or AB homogeneous ) or ( TI “coefficient of variation” or AB “coefficient of variation” ) or ( TI “internal consistency” or AB “internal consistency” ) or (MH “Internal Consistency+”) or (MH “Reliability+”) or (MH “Measurement Error+”) or (MH “Content Validity+”) or “hypothesis testing” or “structural validity” or “cross-cultural validity” or (MH “Criterion-Related Validity+”) or “responsiveness” or “interpretability” or ( TI reliab* or AB reliab* ) and ( (TI test or AB test) OR (TI retest or AB retest) ) or ( TI stability or AB stability ) or ( TI interrater or AB interrater ) or ( TI inter-rater or AB inter-rater ) or ( TI intrarater or AB intrarater ) or ( TI intra-rater or AB intrarater) or ( TI intertester or AB intertester) or (TI inter-tester or AB inter-tester) or ( TI intratester or AB intratester) or ( TI intra-tester or AB intra-tester) or ( TI interobserver or AB interobserver) or (TI inter-observer or AB inter-observer ) or ( TI intraobserver or AB intraobserver) or ( TI intra-observer or AB intra-observer) or ( TI intertechnician or AB intertechnician) or (TI inter-technician or AB inter-techView Resultsnician) or ( TI intratechnician or AB intratechnician ) or ( TI intra-technician or AB intra-technician ) or ( TI interexaminer or AB interexaminer ) or (TI inter-examiner or AB inter-examiner) or (TI intraexaminer or AB intraexaminer ) OR (TI intra-examiner or AB intra-examiner ) or (TI intra-examiner or AB intraexaminer ) or (TI interassay or AB interassay ) or ( TI inter-assay or AB inter-assay ) or ( TI intraassay or AB intraassay) or ( TI intra-assay or AB intra-assay ) or (TI interindividual or AB interindividual) or (TI inter-individual or AB inter-individual) OR (TI intraindividual or AB intraindividual) or (TI intra-individual or AB intra-individual) or (TI interparticipant or AB interparticipant) or (TI inter-participant or AB inter-participant ) or (TI intraparticipant or AB intraparticipant) or (TI intra-participant or AB intra-participant ) or (TI kappa or AB kappa) or (TI kappa’s or AB kappa’s ) or (TI kappas or AB kappas) or (TI repeatab* or AB repeatab*) or ( TI responsive* or AB responsive* ) or ( TI interpretab* or AB interpretab* ) | 778,735 |
| 58 | 56 AND 57 | 296 |

**Cochrane Database of Systematic Reviews & Cochrane Central Register of Controlled Trials via Wiley**

**1996 to 3th April 2024**

4th April 2024

| **#** | **Search Terms** | **Results** |
| --- | --- | --- |
| 1 | MeSH descriptor: [Caregivers] explode all trees | 3,800 |
| 2 | (carer* OR caring OR carergiv* OR care-giv* OR spillover* OR spill over* OR partner* OR husband OR wife OR spouse* OR spousal OR child OR children OR son OR sons OR daughter* OR offspring* OR parent* OR mother* OR maternal OR father* OR paternal OR sibling* OR brother* OR sister* OR dependent* OR “next of kin” OR kinship OR grandparent* OR grandmother* OR grandfather* OR relative* OR family OR “close person” OR families):ti,ab,kw | 440,501 |
| 3 | #5 OR #6 | 441,285 |
| 4 | MeSH descriptor: [Motor Neuron Disease] explode all trees | 1,104 |
| 5 | (ALS OR “Amyotrophic lateral sclerosis” OR “Motor neuron disease” OR “Motor neurone disease” OR MND OR “Charcot’s disease” OR “Charcot disease” OR “anterior horn cell disease” OR “Lateral sclerosis” OR “motor system disease” OR “primary lateral sclerosis” OR PLS OR “progressive muscular atrophy” OR PMA OR “progressive bulbar palsy” OR PBP OR “Lou Gehrig disease”):ti,ab,kw | 4,023 |
| 6 | #4 OR #5 | 4,169 |
| 7 | #3 AND #6 | 981 |
| 8 | (“Acceptance of illness” or ais):ti,ab,kw | 2,414 |
| 9 | (“ALS Depression Inventory 12” or “ALS Depression Inventory” or ADI 12):ti,ab,kw | 98 |
| 10 | (“beck depression inventory” or “beck inventory” or bdi):ti,ab,kw | 7,840 |
| 11 | (“Beck Hopelessness Scale” or bhs):ti,ab,kw | 267 |
| 12 | (“Brief symptom inventory” or bsi):ti,ab,kw | 1,083 |
| 13 | (“burden scale for family caregivers” or bsfc):ti,ab,kw | 18 |
| 14 | (“caregiver burden inventory” or cbi):ti,ab,kw | 381 |
| 15 | (“caregiver burden scale” or cbs):ti,ab,kw | 501 |
| 16 | (“caregiver network scale” or cns):ti,ab,kw | 5,450 |
| 17 | (“caregiver strain index” or csi):ti,ab,kw | 719 |
| 18 | (“carer quality of life” or Carerqol):ti,ab,kw | 94 |
| 19 | (“Center for Epidemiology Articles Depression Scale” or CES-D-10):ti,ab,kw | 40 |
| 20 | (“chalder fatigue scale”):ti,ab,kw | 225 |
| 21 | (“close person questionnaire” or cpq):ti,ab,kw | 50 |
| 22 | (“Coping Inventory for Stressful Situations” or CISS):ti,ab,kw | 133 |
| 23 | (“Coping Orientation to Problems Experienced Inventory” or “brief cope”):ti,ab,kw | 192 |
| 24 | (“cost of care index” or cci):ti,ab,kw | 764 |
| 25 | (“depression anxiety and stress scale” or dass or dass21):ti,ab,kw | 2,481 |
| 26 | (“dyadic adjustment scale” or das):ti,ab,kw | 2,634 |
| 27 | (“euroqol 5 dimensions” or eq5d5l):ti,ab,kw | 766 |
| 28 | (“Folkham's Ways of Coping Scale” or wocq):ti,ab,kw | 4 |
| 29 | (“Functional Assessment of Chronic Illness Therapy Spiritual Well Being Scale” or facit-sp):ti,ab,kw | 117 |
| 30 | (“General Health Questionnaire” or ghq or ghq 12 or ghq12):ti,ab,kw | 1,479 |
| 31 | (“Hamilton Anxiety Rating Scale” or hars):ti,ab,kw | 1,007 |
| 32 | (“Hamilton Depression Rating Scale“ or hdrs):ti,ab,kw | 5,485 |
| 33 | (“Hospital Anxiety and Depression Scale” or hads):ti,ab,kw | 6,746 |
| 34 | (“Life Satisfaction Checklist” or LiSatt11):ti,ab,kw | 16 |
| 35 | (“McGill Quality of Life Questionnaire” or MQol):ti,ab,kw | 63 |
| 36 | (“metacognitive questionnaire 30” or MCQ 30):ti,ab,kw | 88 |
| 37 | (“Multidimensional Scale of Perceived Social Support” or mspss):ti,ab,kw | 168 |
| 38 | (“Patient Health Questionnaire 9” or phq9 or phq 9):ti,ab,kw | 4,692 |
| 39 | (“Positive and Negative Affect Schedule” or panas):ti,ab,kw | 1,002 |
| 40 | (“profile of mood states” or poms or poms sf or “pomssf”):ti,ab,kw | 2,134 |
| 41 | (“purpose in life test” or pil):ti,ab,kw | 166 |
| 42 | (“Quality of Life at the End of Life” or QUAL E fam):ti,ab,kw | 21 |
| 43 | (“Quality of Life Enjoyment & Satisfaction Questionnaire Short Form” or Q-LES-Q-SF):ti,ab,kw | 102 |
| 44 | (“Quality of Life in Life Threatening Illness Family Carer Version 2” or QOLLTI F):ti,ab,kw | 2 |
| 45 | (“Rand 36 Item Health Survey” or rand 36):ti,ab,kw | 584 |
| 46 | (“Satisfaction With Life Scale” or swls):ti,ab,kw | 389 |
| 47 | (“Self Rating Anxiety Scale” or SAS):ti,ab,kw | 5,117 |
| 48 | (“Self Rating Depression Scale” or sds):ti,ab,kw | 4,593 |
| 49 | (“Short Form 12” or “sf 12” or sf12):ti,ab,kw | 4,009 |
| 50 | (“Short Form 36” or “sf 36” or sf36):ti,ab,kw | 18,030 |
| 51 | (“Short Form 8” or “sf 8” or sf8):ti,ab,kw | 358 |
| 52 | (“State-Trait Anxiety Inventory” or staix or staiy or stai):ti,ab,kw | 4,879 |
| 53 | (“The Duke UNC Functional Social Support Questionnaire” or fssq):ti,ab,kw | 10 |
| 54 | (“Ways of Coping Checklist” or wocc):ti,ab,kw | 19 |
| 55 | (“World Health Organization Quality of Life BREF” or whoqol-bref or whoqolbref):ti,ab,kw | 1,478 |
| 56 | (“Zarit Burden Interview” or zbi):ti,ab,kw | 356 |
| 57 | (“Zarit Caregiver Burden Scale”):ti,ab,kw | 36 |
| 58 | (“Zung Depression Scale” or zds):ti,ab,kw | 75 |
| 59 | #8 OR #9 OR #10 OR #11 OR #12 OR #13 OR #14 OR #15 OR #16 OR #17 OR #18 OR #19 OR #20 OR #21 OR #22 OR #23 OR #24 OR #25 OR #26 OR #27 OR #28 OR #29 OR #30 OR #31 OR #32 OR #33 OR #34 OR #35 OR #36 OR #37 OR #38 OR #39 OR #40 OR #41 OR #42 OR #43 OR #44 OR #45 OR #46 OR #47 OR #48 OR #49 OR #50 OR #51 OR #52 OR #53 OR #54 OR #55 OR #56 OR #57 OR #58 | 76,420 |
| 60 | #7 AND #59 | 102 |

**Google Scholar**

22nd April 2024

| 1 | “Acceptance of illness” or ais | 100 |
| --- | --- | --- |
| 2 | “ALS Depression Inventory 12” or “ALS Depression Inventory” or ADI 12 | 36 |
| 3 | “beck depression inventory” or “beck inventory” or bdi | 100 |
| 4 | “Beck Hopelessness Scale” or bhs | 100 |
| 5 | “Brief symptom inventory” or bsi | 100 |
| 6 | “burden scale for family caregivers” or bsfc | 100 |
| 7 | “caregiver burden inventory” or cbi | 100 |
| 8 | “caregiver burden scale” or cbs | 100 |
| 9 | “caregiver network scale” or cns | 1 |
| 10 | “caregiver strain index” or csi | 100 |
| 11 | “carer quality of life” or Carerqol | 100 |
| 12 | “Center for Epidemiology Articles Depression Scale” or CES-D-10 | 35 |
| 13 | “chalder fatigue scale” | 100 |
| 14 | “close person questionnaire” or cpq | 7 |
| 15 | “Coping Inventory for Stressful Situations” or CISS | 100 |
| 16 | “Coping Orientation to Problems Experienced Inventory” or “brief cope” | 100 |
| 17 | “cost of care index” or cci | 100 |
| 18 | “depression anxiety and stress scale” or dass or dass21 | 100 |
| 19 | “dyadic adjustment scale” or das | 100 |
| 20 | “euroqol 5 dimensions” or eq5d5l | 100 |
| 21 | “Folkham's Ways of Coping Scale” or wocq | 0 |
| 22 | “Functional Assessment of Chronic Illness Therapy Spiritual Well Being Scale” or facit-sp | 100 |
| 23 | “General Health Questionnaire” or ghq or ghq 12 or ghq12 | 100 |
| 24 | “Hamilton Anxiety Rating Scale” or hars | 100 |
| 25 | “Hamilton Depression Rating Scale“ or hdrs | 100 |
| 26 | “Hospital Anxiety and Depression Scale” or hads | 100 |
| 27 | “Life Satisfaction Checklist” or LiSatt11 | 0 |
| 28 | “McGill Quality of Life Questionnaire” or MQol | 100 |
| 29 | “metacognitive questionnaire 30” or MCQ 30 | 84 |
| 30 | “Multidimensional Scale of Perceived Social Support” or mspss | 100 |
| 31 | “Patient Health Questionnaire 9” or phq9 or phq 9 | 100 |
| 32 | “Positive and Negative Affect Schedule” or panas | 100 |
| 33 | “profile of mood states” or poms or poms sf or “pomssf” | 11 |
| 34 | “purpose in life test” or pil | 100 |
| 35 | “Quality of Life at the End of Life” or QUAL E fam | 100 |
| 36 | “Quality of Life Enjoyment and Satisfaction Questionnaire Short Form” or Q-LES-Q-SF | 100 |
| 37 | “Quality of Life in Life Threatening Illness Family Carer Version 2” or QOLLTI F | 5 |
| 38 | “Rand 36 Item Health Survey” or rand 36 | 100 |
| 39 | “Satisfaction With Life Scale” or swls | 100 |
| 40 | “Self Rating Anxiety Scale” or SAS | 100 |
| 41 | “Self Rating Depression Scale” or sds | 100 |
| 42 | “Short Form 12” or “sf 12” or sf12 | 100 |
| 43 | “Short Form 36” or “sf 36” or sf36 | 100 |
| 44 | “Short Form 8” or “sf 8” or sf8 | 100 |
| 45 | “State-Trait Anxiety Inventory” or staix or staiy or stai | 100 |
| 46 | “The Duke UNC Functional Social Support Questionnaire” or fssq | 100 |
| 47 | “Ways of Coping Checklist” or wocc | 100 |
| 48 | “World Health Organization Quality of Life BREF” or whoqol-bref or whoqolbref | 100 |
| 49 | “Zarit Burden Interview” or zbi | 100 |
| 50 | “Zarit Caregiver Burden Scale” | 100 |
| 51 | “Zung Depression Scale” or zds | 100 |
