# Supplementary material for: Health-related quality of life of informal carers in ALS: a systematic review of person reported outcome measures
Source: Qual Life Res. 2025 Jun 25;34(10):2731–44. doi: 10.1007/s11136-025-04012-y (PMC12535500; doi:10.1007/s11136-025-04012-y)
Supplement: Supplementary file 4 — Supplementary Material 4 [file 11136_2025_4012_MOESM4_ESM.docx]

**Supplementary Material 4:**

**Table 1. Reference List of Included Articles identified in the review. Table 2. PROM development articles**

**Title:** Health-Related Quality of Life of Informal Carers in ALS: A Systematic Review of Person Reported Outcome Measures

**Journal:** Quality of Life Research

**Authors:** Ms Rosie Bamber, Dr Theocharis Stavroulakis, Professor Christopher McDermott and Professor Jill Carlton

**Corresponding Author:**

Professor Jill Carlton, PhD

Professor of Health Outcomes

Sheffield Centre for Health and Related Research (SCHARR)

University of Sheffield

j.carlton@sheffield.ac.uk

**Table 1. Reference List of Included Articles identified in the review**

(Date Order)

| **AUTHOR** | **DATE** | **TITLE** |
| --- | --- | --- |
| Goldstein, L. H. et al. (1) | 1998 | The psychological impact of MND on patients and carers |
| Ganzini, L. et al. (2) | 1998 | Attitudes of patients with amyotrophic lateral sclerosis and their care givers toward assisted suicide |
| Rabkin, J. G. et al. (3) | 2000 | Resilience and distress among amyotrophic lateral sclerosis patients and caregivers |
| Jenkinson, C. et al. (4) | 2000 | The ALS Health Profile Study: quality of life of amyotrophic lateral sclerosis patients and carers in Europe |
| Hecht, M. J. et al. (5) | 2003 | Burden of care in amyotrophic lateral sclerosis |
| Adelman, E. E. et al. (6) | 2004 | Disparities in perceptions of distress and burden in ALS patients and family caregivers |
| Van den Berg, J. P. et al. (7) | 2005 | Multidisciplinary ALS care improves quality of life in patients with ALS |
| Lo Coco, G. et al. (8) | 2005 | Individual and health-related quality of life assessment in amyotrophic lateral sclerosis patients and their caregivers |
| Chio, A. et al. (9) | 2005 | Caregiver burden and patients' perception of being a burden in ALS |
| Mustfa, N. et al. (10) | 2006 | The effect of non-invasive ventilation on ALS patients and their caregivers |
| Kristjanson, L. J. et al. (11) | 2006 | Palliative care and support for people with neurodegenerative conditions and their carers |
| Gauthier, A. et al. (12) | 2007 | A longitudinal study on quality of life and depression in ALS patient-caregiver couples |
| Vignola, A. et al. (13) | 2008 | Anxiety undermines quality of life in ALS patients and caregivers |
| O'Connor, J. et al. (14) | 2008 | The impact of neurological illness on marital relationships |
| Roach, A. R. et al. (15) | 2009 | The dynamics of quality of life in ALS patients and caregivers |
| Rabkin, J. G. et al. (16) | 2009 | How common is depression among ALS caregivers? A longitudinal study |
| Murphy, V. et al. (17) | 2009 | Problem solving skills predict quality of life and psychological morbidity in ALS caregivers |
| Mockford, C. et al. (18) | 2009 | Development of the Motor Neuron Disease Carer Questionnaire |
| McCabe, M. P. et al. (19) | 2009 | A comparison of mood and quality of life among people with progressive neurological illnesses and their caregivers |
| Palmieri, A. et al (20) | 2009 | Emotional lability in MND: Relationship to cognition and psychopathology and impact on caregivers |
| Olsson, A. G. et al. (21)* | 2010 | Differences in quality-of-life modalities give rise to needs of individual support in patients with ALS and their next of kin |
| Chio, A. et al. (22) | 2010 | Neurobehavioral symptoms in ALS are negatively related to caregivers' burden and quality of life |
| Atkins, L. et al. (23) | 2010 | Marital relationships in amyotrophic lateral sclerosis |
| Pagnini, F. et al. (24) | 2011 | Existential well-being and spirituality of individuals with amyotrophic lateral sclerosis is related to psychological well-being of their caregivers |
| Olsson Ozanne, A. G. et al. (25)* | 2011 | Quality of life, anxiety and depression in ALS patients and their next of kin |
| Ng, L. et al. (26) | 2011 | Patient/carer perceptions of disability in motor neurone disease and carer coping...including commentary by Chio A |
| Calvo, A. et al. (27) | 2011 | Religiousness is positively associated with quality of life of ALS caregivers |
| Peters, M. et al. (28) | 2012 | The impact of perceived lack of support provided by health and social care services to caregivers of people with motor neuron disease |
| Marconi, A. et al. (29) | 2012 | Aggressiveness, sexuality, and obsessiveness in late stages of ALS patients and their effects on caregivers |
| Lillo, P. et al. (30) | 2012 | Caregiver burden in amyotrophic lateral sclerosis is more dependent on patients' behavioral changes than physical disability: a comparative study |
| Joubert, K. et al. (31) | 2012 | Motor neuron disease: The impact of decreased speech intelligibility on marital communication |
| Boerner, K. et al. (32) | 2012 | Impact of patient suffering on caregiver well-being: The case of amyotrophic lateral sclerosis patients and their caregivers |
| Peters, M. et al. (33) | 2013 | Carer quality of life and experiences of health services: a cross-sectional survey across three neurological conditions |
| Baxter, S. K. et al. (34) | 2013 | The impact on the family carer of motor neurone disease and intervention with non-invasive ventilation |
| Tramonti, F. et al. (35) | 2014 | A permanent emergency: a longitudinal study on families coping with amyotrophic lateral sclerosis |
| Qutub, K. et al. (36) | 2014 | Life factors affecting depression and burden in amyotrophic lateral sclerosis caregivers |
| Kim, C. H. et al. (37) | 2014 | Ventilator use, respiratory problems, and caregiver well-being in Korean patients with amyotrophic lateral sclerosis receiving home-based care |
| Hwang, C. S. et al. (38) | 2014 | An eye-tracking assistive device improves the quality of life for ALS patients and reduces the caregivers' burden |
| Creemers, H. et al. (39) | 2014 | Cluster RCT of case management on patients' quality of life and caregiver strain in ALS |
| Watermeyer, T. J. et al. (40) | 2015 | Impact of disease, cognitive and behavioural factors on caregiver outcome in amyotrophic lateral sclerosis |
| Van Groenestijn, A. C. et al. (41) | 2015 | Cognitive behavioural therapy and quality of life in psychologically distressed patients with amyotrophic lateral sclerosis and their caregivers: results of a prematurely stopped randomized controlled trial |
| Londral, A. et al. (42) | 2015 | Quality of life in amyotrophic lateral sclerosis patients and caregivers: Impact of assistive communication from early stages |
| Kleinbub, J. R. et al. (43) | 2015 | Hypnosis-based psychodynamic treatment in ALS: A longitudinal study on patients and their caregivers |
| Cui, B. et al. (44) | 2015 | Behavioral Symptoms in Motor Neuron Disease and Their Negative Impact on Caregiver Burden |
| Burke, T. et al. (45) | 2015 | Caregiver burden in amyotrophic lateral sclerosis: a cross-sectional investigation of predictors |
| Tramonti, F. et al (46) | 2015 | Age, gender, kinship and caregiver burden in amyotrophic lateral sclerosis |
| Aoun, S. M. et al. (47) | 2015 | Dignity therapy for people with motor neuron disease and their family caregivers: A feasibility study |
| Tremolizzo, L. et al. (48) | 2016 | Behavioural But Not Cognitive Impairment Is a Determinant of Caregiver Burden in Amyotrophic Lateral Sclerosis |
| Steinhauser, K. E. et al. (49) | 2016 | The feasibility and acceptability of a chaplain-led intervention for caregivers of seriously ill patients: A Caregiver Outlook pilot study |
| Pagnini, F. et al. (50) | 2016 | Mindfulness as a Protective Factor for the Burden of Caregivers of Amyotrophic Lateral Sclerosis Patients |
| Kurien, M. et al. (51) | 2016 | Gastrostomies Preserve But Do Not Increase Quality of Life for Patients and Caregivers |
| Galvin, M. et al. (52) | 2016 | Caregiving in ALS - a mixed methods approach to the study of Burden |
| Creemers, H. et al. (53) | 2016 | Factors related to caregiver strain in ALS: a longitudinal study |
| Hsieh, S. et al. (54) | 2016 | The Evolution of Caregiver Burden in Frontotemporal Dementia with and without Amyotrophic Lateral Sclerosis |
| Veronese, S. et al. (55) | 2017 | Specialist palliative care improves the quality of life in advanced neurodegenerative disorders: NE-PAL, a pilot randomised controlled study |
| Siciliano, M. et al. (56) | 2017 | Coping strategies and psychological distress in caregivers of patients with Amyotrophic Lateral Sclerosis (ALS) |
| Johnson, S. et al. (57) | 2017 | Quality of Life Perspectives of People With Amyotrophic Lateral Sclerosis and Their Caregivers |
| Geng, D. et al. (58) | 2017 | Patients' self-perceived burden, caregivers' burden and quality of life for amyotrophic lateral sclerosis patients: a cross-sectional study |
| Burke, T. et al. (59) | 2017 | Caregivers of patients with amyotrophic lateral sclerosis: investigating quality of life, caregiver burden, service engagement, and patient survival |
| Thomas, P. T. et al. (60) | 2018 | Caregiver burden and quality of life of patients with amyotrophic lateral sclerosis in India |
| Sandstedt, P. et al. (61) | 2018 | Caregiver experience, health-related quality of life and life satisfaction among informal caregivers to patients with amyotrophic lateral sclerosis: A cross-sectional study |
| Quattropani, M. C. et al. (62) | 2018 | Emotional burden and coping strategies in amyotrophic lateral sclerosis caregivers: The role of metacognitions |
| Oh, J. et al. (63) | 2018 | Factor analysis of the Zarit Burden Interview in family caregivers of patients with amyotrophic lateral sclerosis |
| Galvin, M. et al. (64) | 2018 | Needs of informal caregivers across the caregiving course in amyotrophic lateral sclerosis: a qualitative analysis |
| Burke, T. et al. (65) | 2018 | Longitudinal predictors of caregiver burden in amyotrophic lateral sclerosis: a population-based cohort of patient-caregiver dyads |
| Hobson, E. V. et al. (66) | 2019 | Using telehealth in motor neuron disease to increase access to specialist multidisciplinary care: a UK-based pilot and feasibility study |
| Tramonti, F. et al. (67) | 2019 | Caregiver burden and family functioning in different neurological diseases |
| De Wit, J. et al. (68) | 2019 | Psychological distress and coping styles of caregivers of patients with amyotrophic lateral sclerosis: a longitudinal study |
| Galvin, M. et al. (69) | 2020 | Individual quality of life in spousal ALS patient-caregiver dyads |
| De Wit, J. et al. (70) | 2020 | Blended psychosocial support for partners of patients with ALS and PMA: results of a randomized controlled trial |
| Tang, S. et al. (71) | 2021 | Caregiver burden and associated factors among primary caregivers of patients with ALS in home care: a cross-sectional survey study |
| Conroy, E. et al. (72) | 2021 | Informal Caregivers in Amyotrophic Lateral Sclerosis: A Multi-Centre, Exploratory Study of Burden and Difficulties |
| Antoniadi, A. M. et al. (73) | 2021 | Prediction of caregiver quality of life in amyotrophic lateral sclerosis using explainable machine learning |
| Pagnini, F. et al. (74) | 2022 | An online non-meditative mindfulness intervention for people with ALS and their caregivers: a randomized controlled trial |
| Larsson, B. J. et al. (75) | 2022 | Quality of life among relatives of patients with amyotrophic lateral sclerosis: A prospective and longitudinal study |
| Kennedy, P. et al. (76) | 2022 | Burden and benefit-A mixed methods study of informal Amyotrophic Lateral Sclerosis caregivers in Ireland and the Netherlands |
| Giusiano, S. et al. (77) | 2022 | Amyotrophic lateral sclerosis caregiver burden and patients' quality of life during COVID-19 pandemic |
| De Sousa, M. J. V. et al. (78) | 2022 | Burden of informal caregivers of people with amyotrophic lateral sclerosis |
| Sharbafshaaer, M. et al. (79) | 2022 | Psychological Support for Family Caregivers of Patients with Amyotrophic Lateral Sclerosis at the Time of the Coronavirus Disease 2019 Pandemic: A Pilot Study Using a Telemedicine Approach |
| Beaton, D. et al. (80) | 2022 | Caregiving concerns and clinical characteristics across neurodegenerative and cerebrovascular disorders in the Ontario neurodegenerative disease research initiative |
| Maksymowicz-Sliwinska, A. et al. (81) | 2023 | The quality of life and depression in primary caregivers of patients with amyotrophic lateral sclerosis is affected by patient-related and culture-specific conditions |
| Gentili, D. et al. (82) | 2023 | Quality of Life in Amyotrophic Lateral Sclerosis Patients and Care Burden of Caregivers in Sardinia during COVID-19 Pandemic |
| Conroy, E. et al. (83) | 2023 | Care, burden and self-described positive aspects of caring in amyotrophic lateral sclerosis: an exploratory, longitudinal, mixed-methods study |
| Wei, Q. Q. et al. (84) | 2023 | Prevalence and associated factors of apathy in Chinese ALS patients |
| Tulek, Z. et al. (85) | 2023 | Care burden and related factors among informal caregivers of patients with amyotrophic lateral sclerosis |

| Table 1: Articles that use HRQoL PROMs with adult informal carers of people with ALS  *Indicates articles reporting on the same dataset. |
| --- |

**Table 2. PROM development articles**

(Alphabetical According to PROM Abbreviation)

Note: Two development articles were used to assess the following PROMs: Coping Orientation to Problems Experienced Inventory (Brief COPE), Functional Assessment of Chronic Illness Therapy–Spiritual Well-Being Scale (Facit-Sp), Metacognitive Questionnaire (MCQ-30) and Profile of Mood States - Short Form (POMS-SF).

| **PROM Abbreviation** | **Author** | **Year** | **Development Article Title** |
| --- | --- | --- | --- |
| AIS | Felton, B. J. et al. (1) | 1984 | Coping with chronic illness: A study of illness controllability and the influence of coping strategies on psychological adjustment |
| BDI | Beck, A. T. et al. (2) | 1961 | An inventory for measuring depression |
| BHS | Beck, A. T. et al (3) | 1974 | The measurement of pessimism: the pessimism: the hopelessness scale |
| Brief COPE | Carver, C. S. (4) | 1997 | You want to measure coping but your protocol's too long: consider the brief COPE |
| Brief COPE | Carver, C. S. et al. (5) | 1989 | Assessing coping strategies: a theoretically based approach |
| BSFC | Grasel, E. (6) | 1995 | Somatic symptoms and caregiving strain among family caregivers of older patients with progressive nursing needs |
| CarerQoL | Brouwer, W. et al. (7) | 2006 | The CarerQol instrument: a new instrument to measure care-related quality of life of informal caregivers for use in economic evaluations |
| CBI | Novak, M. et al. (8) | 1989 | Application of a multidimensional caregiver burden inventory |
| CES-D-10 | Radloff, L. S. (9) | 1977 | The CES-D Scale: A self-report depression scale for research in the general population |
| CFS | Chalder, T. et al. (10) | 1993 | Development of a fatigue scale |
| CFS Physical Subscale | Chalder, T. et al. (10) | 1993 | Development of a fatigue scale |
| CNS | Love, A. et al. (11) | 2004 | Social aspects of caregiving for people living with motor neurone disease: their relationships to carer well-being |
| CPQ | Stansfeld, S. et al. (12) | 1992 | Deriving a survey measure of social support: the reliability and validity of the Close Persons Questionnaire |
| CSI | Robinson, B.C. (13) | 1983 | Validation of a caregiver strain index |
| DAS | Spanier, G. et al. (14) | 1976 | Measuring dyadic adjustment: new scales for assessing the quality of marriage and similar dyads |
| EQ-5D-5L | Herdman, M. et al. (15) | 2011 | Development and preliminary testing of the new five-level version of EQ-5D (EQ-5D-5L) |
| EWBS | Cohen, S. R. et al. (16) | 1995 | The McGill Quality of Life questionnaire: a measure of quality of life appropriate for people with advanced diseases. A preliminary study of validity and acceptability |
| FACIT-Sp | Cella, D. F. et al. (17) | 1993 | The Functional Assessment of Cancer Therapy scale: development and validation of the general measure |
| FACIT-Sp | Peterman, A. H. et al. (18) | 2002 | Measuring spiritual well-being in people with cancer: the functional assessment of chronic illness therapy--Spiritual Well-being Scale (FACIT-Sp) |
| FSSQ | Broadhead, W. E. et al. (19) | 1988 | The Duke-UNC Functional Social Support Questionnaire. Measurement of social support in family medicine patients |
| GHQ | Goldberg, D. (20) | 1979 | The detection of psychiatric illness by questionnaire : a technique for the identification and assessment of non-psychotic psychiatric illness |
| HADS | Zigmoid, A. S. et al. (21) | 1983 | The hospital anxiety and depression scale |
| LiSat-11 | Fugl-Meyer, A. R. et al. (22) | 1991 | Happiness and domain-specific life satisfaction in adult northern Swedes |
| MCQ-30 | Wells, A. et al. (23) | 2004 | A short form of the metacognitions questionnaire: properties of the MCQ-30 |
| MCQ-65 | Cartwright-Hatton, S. et al. (24) | 1997 | Beliefs about worry and intrusions: the Meta-Cognitions Questionnaire and its correlates |
| MPSS | Zimet, G.D. et al. (25) | 1988 | The multidimensional scale of perceived social support |
| MQOL | Cohen, S. R. et al. (16) | 1995 | The McGill Quality of Life questionnaire: a measure of quality of life appropriate for people with advanced diseases. A preliminary study of validity and acceptability |
| PANAS | Watsin, D. et al. (26) | 1988 | Development and validation of brief measures of positive and negative affect: the PANAS scales |
| PHQ-9 | Spitzer, R. L. (27) | 1999 | Validation and Utility of a Self-report Version of PRIME-MD The PHQ Primary Care Study |
| POMS-SF | Curran, S. L. et al. (28) | 1995 | Short form of the Profile of Mood States (POMS-SF): Psychometric information |
| POMS-SF | Shacham, S. (29) | 1983 | A Shortened Version of the Profile of Mood States. Journal of Personality Assessment |
| Q-LES-Q-SF | Endicott, J. et al. (30) | 1993 | Quality of Life Enjoyment and Satisfaction Questionnaire: a new measure |
| QOLLTI-F | Cohen, R. et al. (31) | 2006 | QOLLTI-F: measuring family carer quality of life |
| QUAL-E (Fam) | Steinhauser, K.E. et al. (32) | 2014 | Initial Validation of a New Instrument to Measure Quality of Life at the End of Life (QUAL-E) |
| RAND-36 | Hays, R. D. et al. (33) | 1993 | The RAND 36-Item Health Survey 1.0 |
| SAS | Zung, W. W. et al. (34) | 1971 | A rating instrument for anxiety disorders |
| SDS | Zung, W.W. et al. (35) | 1965 | A self-rating depression scale |
| SF-12 | Ware, J. et al. (36) | 2002 | User’s manual for the SF-12v2 Health Survey |
| SF-36 | Ware, J. et al. (37) | 1992 | The MOS 36-item short form health survey (SF-36): Conceptual framework and items selection |
| SF-36 MCS | Ware, J. et al. (37) | 1992 | The MOS 36-item short form health survey (SF-36): Conceptual framework and items selection |
| SF-36 V2 | Ware, J. et al. (38) | 2000 | How to score version 2 of the SF36 health survey |
| STAI-X | Spielberger, C. D. et al. (39) | 1970 | The State-Trait Anxiety Inventory |
| STAI-Y1 | Spielberger, C. D. et al. (40) | 1983 | Manual for the State-Trait Anxiety Inventory |
| STAI-Y1 & Y2 | Spielberger, C. D. et al. (40) | 1983 | Manual for the State-Trait Anxiety Inventory |
| SWLS | Diener, E. et al. (41) | 1985 | The Satisfaction with Life Scale. Journal of Personality Assessment |
| WHOQOL-BREF | The WHOQOL Group. (42) | 1998 | Development of the World Health Organiza-tion WHOQOL-BREF quality of life assessment |
| ZBI | Zarit, S.H. et al. (43) | 1980 | Relatives of the impaired elderly: correlates of feelings of burden |
