# Supplementary material for: Health-related quality of life of informal carers in ALS: a systematic review of person reported outcome measures
Source: Qual Life Res. 2025 Jun 25;34(10):2731–44. doi: 10.1007/s11136-025-04012-y (PMC12535500; doi:10.1007/s11136-025-04012-y)
Supplement: Supplementary file 5 — Supplementary Material 5 [file 11136_2025_4012_MOESM5_ESM.docx]

**Supplementary Material 5: Full List of PROMs Identified in Searches**

**Title:** Health-Related Quality of Life of Informal Carers in ALS: A Systematic Review of Person Reported Outcome Measures

**Journal:** Quality of Life Research

**Authors:** Ms Rosie Bamber, Dr Theocharis Stavroulakis, Professor Christopher McDermott and Professor Jill Carlton

**Corresponding Author:**

Professor Jill Carlton, PhD

Professor of Health Outcomes

Sheffield Centre for Health and Related Research (SCHARR)

University of Sheffield

j.carlton@sheffield.ac.uk

|  | **PROM Name** | **Abbreviation** | **Reviewed (Y/N)** | **Exclusion Rationale** |
| --- | --- | --- | --- | --- |
| 1 | Amnestic Comparative Self-Assessment | ACSA | Y | 0 |
| 2 | ALS Depression Inventory-12 Item | ADI-12 | Y | 7 |
| 3 | Acceptance of Illness Scale | AIS | Y | 8 |
| 4 | ALS Cognitive Behavioural Screen | ALS CBS | Y | 4 |
| 5 | ALS Caregiver Needs and Burden Questionnaire | ALS CNB-Q | Y | 4 |
| 6 | Caregiver Burden Scale – ALS Patient Caregiver Form | ALSPCF | N | N/A |
| 7 | Affiliate Stigma Scale | ASS | Y | 6 |
| 8 | Beck Depression Inventory | BDI | Y | 8 |
| 9 | Beck Depression Inventory-II | BDI-II | Y | 7 |
| 10 | Big Five Questionnaire | BFQ | N | N/A |
| 11 | Beck Hopelessness Scale | BHS | Y | 8 |
| 12 | Coping Orientation to Problems Experienced Inventory | Brief COPE | Y | 8 |
| 13 | Burden Scale for Family Caregivers | BSFC | Y | 8 |
| 14 | Brief Symptom Inventory | BSI | Y | 7 |
| 15 | Carer Quality of Life | CarerQoL | Y | 8 |
| 16 | Caregiver Burden Inventory | CBI | Y | 8 |
| 17 | Caregiver Burden Scale | CBS | Y | 7 |
| 18 | Cost of Care Index | CCI | Y | 7 |
| 19 | Center for Epidemiology Articles Depression Scale | CES-D-10 | Y | 8 |
| 20 | Modified Communication Effectiveness Index | CETI-M | Y | 5 |
| 21 | Chalder Fatigue Scale | CFS | Y | 8 |
| 22 | Chalder Fatigue Scale - Physical Fatigue Subscale | CFS - Physical | Y | 8 |
| 23 | Coping Inventory for Stressful Situations | CISS | Y | 7 |
| 24 | Caregiver Network Scale | CNS | Y | 8 |
| 25 | Close Persons Questionnaire | CPQ | Y | 8 |
| 26 | Caregiver Reaction Assessment | CRA | N | N/A |
| 27 | Caregiver Strain Index | CSI | Y | 8 |
| 28 | Caregiver Strain Scale | CSS | N | N/A |
| 29 | Dyadic Adjustment Scale - Dyadic Subscale | DAS | Y | 8 |
| 30 | Depression, Anxiety Stress Scale | DASS-21 | Y | 7 |
| 31 | Diagnostic Interview Schedule | DIS | N | N/A |
| 32 | Daily Spiritual Experience Scale | DSES | N | 5 |
| 33 | Duke University Religion Index | DUREL | N | 5 |
| 34 | EuroQoL-5 Dimensions | EQ-5D-5L | Y | 8 |
| 35 | EuroQoL Visual Analogue Scale | EQ-5D-5L VAS | Y | 0 |
| 36 | Existential Well-Being Subscale from the McGill Quality of Life Questionnaire | EWBS | Y | 8 |
| 37 | Family Cohesions and Adaptability III | FACES III | Y | 5 |
| 38 | Functional Assessment of Chronic Illness Therapy–Spiritual Well-Being Scale | FACIT-Sp | Y | 8 |
| 39 | Family Satisfaction with Care | FAMCARE | Y | 5 |
| 40 | Folkham's Measure of Caregiver Satisfaction | Folkham's | N | N/A |
| 41 | Folkham's 4-item Measure of Finding Positive Meaning in Caregiving | Folkham's 4-Item | N | N/A |
| 42 | The Duke-UNC Functional Social Support Questionnaire | FSSQ | Y | 8 |
| 43 | General Health Questionnaire | GHQ | Y | 8 |
| 44 | General Health Questionnaire-12 | GHQ-12 | Y | 7 |
| 45 | Hospital Anxiety & Depression Scale | HADS | Y | 8 |
| 46 | Hamilton Anxiety Rating Scale | HARS | Y | 3 |
| 47 | Hamilton Depression Rating Scale | HDRS | Y | 3 |
| 48 | Idler Index of Religiosity | IIR | Y | 5 |
| 49 | Job Content Questionnaire | JCQ | Y | 5 |
| 50 | Life Satisfaction Checklist | LiSat-11 | Y | 8 |
| 51 | Langer Mindfulness Scale | LMS | Y | 5 |
| 52 | The Level of Care Index | LoC Index | N | N/A |
| 53 | The Life Rating Scale | LRS | N | N/A |
| 54 | Manne Scales of Positive and Negative Dyad Support | Manne Scales | N | N/A |
| 55 | Metacognitive Questionnaire 30 | MCQ-30 | Y | 8 |
| 56 | Marital Intimacy Scale | MIS | Y | 5 |
| 57 | Munich Quality of Life Dimensions List | MLDL | N | 1 |
| 58 | Motor Neuron Disease Care Satisfaction | MND Care | N | N/A |
| 59 | Motor Neuron Disease Carer Questionnaire | MNDCQ | N | N/A |
| 60 | Multidimensional Scale of Perceived Social Support | MPSS | Y | 8 |
| 61 | McGill Quality of Life Questionnaire | MQOL | Y | 8 |
| 62 | McGill Quality of Life Questionnaire Single-Item Scale | MQOL-SIS | Y | 0 |
| 63 | Positive and Negative Affect Schedule | PANAS | Y | 8 |
| 64 | Primary Communication Inventory | PCI | N | N/A |
| 65 | Prolonged Grief Scale | PG-12 | Y | 3 |
| 66 | Patient Health Questionnaire-9 | PHQ-9 | Y | 8 |
| 67 | Purpose in Life Test | PIL | Y | 7 |
| 68 | Profile of Mood States - 11 Item | POMS 11-Item | N | 2 |
| 69 | Profile of Mood States - 65 Item | POMS 65-Item | N | 2 |
| 70 | Profile of Mood States - Short Form | POMS-SF | Y | 8 |
| 71 | QoL Enjoyment & Satisfaction Questionnaire Short Form | Q-LES-Q-SF | Y | 8 |
| 72 | Informal Caregiver Burden Assessment Questionnaire | QASCI | Y | 7 |
| 73 | Quality of Care | QoC | N | N/A |
| 74 | Quality of Life Index | QoL Index | N | N/A |
| 75 | Quality of Life Inventory | QoL Inventory | N | N/A |
| 76 | Quality of Life in Life-Threatening Illness Family Carer Version | QOLLTI-F | Y | 8 |
| 77 | Quality of Relationships Inventory | QRI | Y | 5 |
| 78 | Quality of Life at the End of Life | QUAL-E (fam) | Y | 8 |
| 79 | Rand 36-Item Health Survey | RAND-36 | Y | 8 |
| 80 | Relationship Assessment Scale | RAS | Y | 5 |
| 81 | Brief Religious Scoping Activity Scale | RCOPE | Y | 5 |
| 82 | Revised Scale for Caregiving Self-Efficacy - Controlling Upsetting Thoughts Subscale | RSCSE-Contr | Y | 5 |
| 83 | Revised Scale for Caregiving Self-Efficacy | RSCSE-Resp | Y | 5 |
| 84 | Relationship Satisfaction Scale | RSS | N | N/A |
| 85 | Self-Rating Anxiety Scale | SAS | Y | 8 |
| 86 | Systems of Belief Inventory | SBI-15R | Y | 5 |
| 87 | Self-Rating Depression Scale | SDS | Y | 8 |
| 88 | Schedule for the Evaluation of Individual Quality of Life–Direct Weight | SEIQoL-DW | Y | 3 |
| 89 | Short Form-12 | SF-12 | Y | 8 |
| 90 | Short Form-36 | SF-36 | Y | 8 |
| 91 | Short Form-36 Mental Component Summary | SF-36 MCS | Y | 8 |
| 92 | Short Form-36 Version 2 | SF-36 V2 | Y | 8 |
| 93 | Short Form-8 | SF-8 | Y | 7 |
| 94 | Single Item Scale - Religiosity & Spirituality | SIS Religiosity | N | N/A |
| 95 | The Spiritual Perspective Scale | SPS | Y | 5 |
| 96 | Social Problem-Solving Inventory Revised | SPSI-R | N | N/A |
| 97 | Self-Rated Burden | SRB | Y | 0 |
| 98 | Social Supports Questionnaire-6 | SSQ-6 | Y | 5 |
| 99 | Sexuality Self-Reporting Scale | SSRS | N | N/A |
| 100 | State-Trait Anxiety Inventory-X | STAI-X | Y | 8 |
| 101 | State-Trait Anxiety Inventory-Y1 | STAI-Y1 | Y | 8 |
| 102 | State-Trait Anxiety Inventory-Y | STAI-Y1 & Y2 | Y | 8 |
| 103 | Satisfaction With Life Scale | SWLS | Y | 8 |
| 104 | Utrecht Coping List | UCL | N | N/A |
| 105 | Visual Analogue Scale for Carer Burden | VAS - Carer Burden | N | N/A |
| 106 | Visual Analogue Scale for Physical, General and Psychological Well-being | VAS - Wellbeing | N | N/A |
| 107 | World Health Organisation Quality of Life-BREF | WHOQOL-BREF | Y | 8 |
| 108 | Ways of Coping Questionnaire | WOCQ | Y | 7 |
| 109 | Zarit Burden Interview | ZBI | Y | 8 |

| **Supplementary Material 5: Full List of Extracted Instruments Identified in Stage 1 Searches**  Table illustrating all 109 PROMs extracted from articles yielded from **Search 1** with full names and abbreviations. Reason for PROM exclusion: (0) Not a multi-item PROM, (1) Not available in English, (2) Not freely available, (3) Not a self-report measure and cannot be used systematically across a population, (4) Does not yield quantitative score/s, (5) 1≤ item covers an aspect of HRQoL, (6) Not a validated version or adaptation of an existing PROM, (7) Development article unsourced or not available in English, (8) Include PROM in next stage of COSMIN review.  Several PROMs remained unreviewed as they remained unsourced despite various methods to locate them, including: (1) Sourcing the PROM development article referenced in-text, (2) Google searches for the name of PROM +/- the term ‘pdf’, (3) Searches and requests via E-Provide database, (4) Author emails sent between 19/02/2024 - 01/03/2024, (5) Call-outs within the University of Sheffield Centre for Health and Related Research and Institute of Translational Neuroscience.  Abb. = Abbreviation, N = No, PROM = Person Reported Outcome Measure, Y = Yes. |
| --- |
