# Supplementary material for: Health-related quality of life of informal carers in ALS: a systematic review of person reported outcome measures
Source: Qual Life Res. 2025 Jun 25;34(10):2731–44. doi: 10.1007/s11136-025-04012-y (PMC12535500; doi:10.1007/s11136-025-04012-y)
Supplement: Supplementary file 6 — Supplementary Material 6 [file 11136_2025_4012_MOESM6_ESM.docx]

**Supplementary Material 6: Frequency of Use of PROMs**

**Title:** Health-Related Quality of Life of Informal Carers in ALS: A Systematic Review of Person Reported Outcome Measures

**Journal:** Quality of Life Research

**Authors:** Ms Rosie Bamber, Dr Theocharis Stavroulakis, Professor Christopher McDermott and Professor Jill Carlton

**Corresponding Author:**

Professor Jill Carlton, PhD

Professor of Health Outcomes

Sheffield Centre for Health and Related Research (SCHARR)

University of Sheffield

j.carlton@sheffield.ac.uk


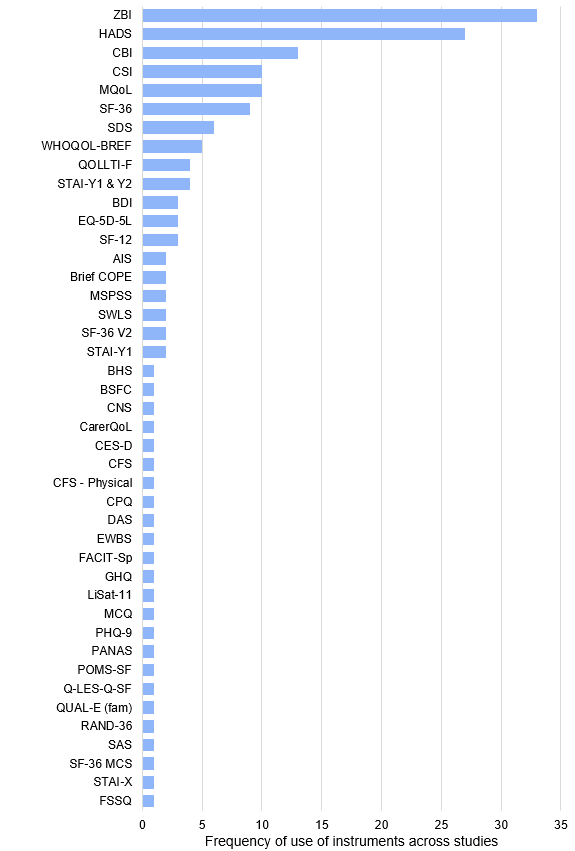


| **Supplementary Material 6: Frequency of Use of PROMs**  Summary and an assessment of the development articles for the 43 PROMs used to assess ALS carer HRQoL from full-text articles meeting eligibility criteria within the Stage 1 search.  PROM Abbreviations: AIS = Acceptance of Illness Scale, BDI = Beck Depression Inventory, BHS = Beck Hopelessness Scale, Brief COPE = Coping Orientation to Problems Experienced Inventory, BSFC = Burden Scale for Family Caregivers, CarerQoL = Carer Quality of Life, CBI = Caregiver Burden Inventory, CES-D-10 = Center for Epidemiology Articles Depression Scale, CFS = Chalder Fatigue Scale, CFS-Physical = Chalder Fatigue Scale - Physical Fatigue Subscale, CNS = Caregiver Network Scale, CPQ = Close Persons Questionnaire, CSI = Caregiver Strain Index, DAS = Dyadic Adjustment Scale - Dyadic Subscale, EQ-5D-5L = EuroQoL-5 Dimensions, EWBS = Existential Well-Being Subscale from the McGill Quality of Life Questionnaire, FACIT-Sp = Functional Assessment of Chronic Illness Therapy–Spiritual Well-Being Scale, FSSQ = The Duke-UNC Functional Social Support Questionnaire, GHQ = General Health Questionnaire, HADS = Hospital Anxiety & Depression Scale, LiSat-11 = Life Satisfaction Checklist, MCQ-30 = Metacognitive Questionnaire 30, MPSS = Multidimensional Scale of Perceived Social Support, MQOL = McGill Quality of Life Questionnaire, PANAS = Positive and Negative Affect Schedule, PHQ-9 = Patient Health Questionnaire-9, POMS-SF = Profile of Mood States - Short Form, Q-LES-Q-SF = QoL Enjoyment & Satisfaction Questionnaire Short Form, QOLLTI-F = Quality of Life in Life-Threatening Illness Family Carer Version, QUAL-E (Fam) = Quality of Life at the End of Life, RAND-36 = Rand 36-Item Health Survey, SAS = Self-Rating Anxiety Scale, SDS = Self-Rating Depression Scale, SF-12 = Short Form-12, SF-36 = Short Form-36, SF-36 MCS = Short Form-36 Mental Component Summary, SF-36 V2 = Short Form-36 Version 2, STAI-X = State-Trait Anxiety Inventory-X, STAI-Y1 = State-Trait Anxiety Inventory-Y1, STAI-Y1 & Y2 = State-Trait Anxiety Inventory-Y, SWLS = Satisfaction With Life Scale, WHOQOL-BREF = World health organisation quality of life-BREF, ZBI = Zarit Burden Interview |
| --- |
